# Gut Microbiota Modulation by Inulin Improves Metabolism and Ovarian Function in Polycystic Ovary Syndrome

**DOI:** 10.1002/advs.202412558

**Published:** 2025-04-07

**Authors:** Lulu Geng, Xin Yang, Jiani Sun, Ximing Ran, Dan Zhou, Mingming Ye, Li Wen, Ruirui Wang, Miaoxin Chen

**Affiliations:** ^1^ Centre for Assisted Reproduction Shanghai Key Laboratory of Maternal‐Fetal Medicine Shanghai Institute of Maternal‐Fetal Medicine and Gynecologic Oncology Shanghai First Maternity and Infant Hospital School of Medicine Tongji University Shanghai 200092 China; ^2^ Shanghai Innovation Center of TCM Health Service Shanghai University of Traditional Chinese Medicine Shanghai 201203 China; ^3^ Section of Endocrinology, Internal Medicine School of Medicine Yale University New Haven CT 06520 USA; ^4^ Department of Biostatistics and Bioinformatics Rollins School of Public Health Emory University Atlanta GA 30322 USA; ^5^ State Key Laboratory of Integration and Innovation of Classic Formula and Modern Chinese Medicine Shanghai University of Traditional Chinese Medicine Shanghai 201203 China

**Keywords:** fecal microbiota transplantation, gut microbiota, inulin, polycystic ovary syndrome, short‐chain fatty acids

## Abstract

The management of metabolic disorder associated with polycystic ovary syndrome (PCOS) has been suggested as an effective approach to improve PCOS which is highly involved with gut microbiota, while the underlying mechanism is unclear. Here, we investigated the role of inulin, a gut microbiota regulator, in the alleviation of PCOS. Our findings showed that inulin treatment significantly improved hyperandrogenism and glucolipid metabolism in both PCOS cohort and mice. Consistent with the cohort, inulin increased the abundance of microbial co‐abundance group (CAG) 12 in PCOS mice, including *Bifidobacterium* species and other short‐chain fatty acids (SCFAs)‐producers. We further verified the enhancement of SCFAs biosynthesis capacity and fecal SCFAs content by inulin. Moreover, inulin decreased lipopolysaccharide‐binding protein (LBP) and ameliorated ovarian inflammation in PCOS mice, whereas intraperitoneal lipopolysaccharide (LPS) administration reversed the protective effects of inulin. Furthermore, fecal microbiota transplantation (FMT) from inulin‐treated patients with PCOS enhanced insulin sensitivity, improved lipid accumulation and thermogenesis, reduced hyperandrogenism and ovarian inflammatory response in antibiotic‐treated mice. Collectively, these findings revealed that gut microbiota mediates the beneficial effects of inulin on metabolic disorder and ovarian dysfunction in PCOS. Therefore, modulating gut microbiota represents a promising therapeutic strategy for PCOS.

## Graphical abstract

This work proposes a novel strategy targeting gut microbiota for managing polycystic ovary syndrome (PCOS). Inulin, a gut microbiota modulator, notably boosts the growth of short‐chain fatty acids (SCFAs)‐producers, represented by *Bifidobacterium*, thereby improving glucolipid metabolism and ovarian function of PCOS through suppressing the inflammatory responses.

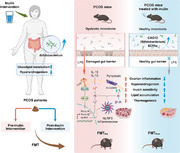

## 1 Introduction

Polycystic ovary syndrome (PCOS) is the most prevalent reproductive endocrine disorder among women of reproductive age, with a global prevalence estimated between 5% and 20%.^[^ ^1^, ^2^ ^]^ The primary clinical presentations of PCOS include hyperandrogenism, irregular menstruation, oligomenorrhea, and anovulation.^[^ ^3^ ^]^ In addition to reproductive issues, PCOS is often accompanied by metabolic complications, which interrelate with reproductive abnormalities and exacerbate pathological progression.^[^ ^4^, ^5^, ^6^, ^7^ ^]^ Notably, immune response and metabolic regulation are closely interconnected,^[^ ^8^ ^]^ and low‐grade chronic inflammation has been strongly linked to metabolic disorders, including PCOS.^[^ ^6^ ^]^ Currently, there is still no effective therapy for PCOS. Lifestyle management serves as the first‐line treatment in all patients with PCOS for improving metabolic health,^[^ ^9^ ^]^ which provides possibly reproductive benefits.^[^ ^10^, ^11^, ^12^ ^]^ However, the underlying mechanism remains unclear.

The etiology of PCOS is considered multifactorial, involving genetic, epigenetic, and environmental factors that collectively contribute to its onset and progression.^[^ ^1^ ^]^ Increasing evidence suggests that women with PCOS exhibit gut microbial dysbiosis, characterized by decreased alpha diversity, altered beta diversity, and an imbalance in the Firmicutes‐to‐Bacterioidetes ratio.^[^ ^13^, ^14^, ^15^ ^]^ A consistent pattern has emerged showing a decrease in beneficial probiotics and an increase in opportunistic pathogens in the gut microbiota of women with PCOS.^[^ ^14^, ^15^, ^16^, ^17^, ^18^ ^]^ Animal studies further support the role of gut microbiota as a causative factor in PCOS. For instance, transplanting gut microbiota or even a single pathogenic strain from women with PCOS into recipient mice can induce reproductive and metabolic phenotypes of PCOS, accompanied by disruptions in bile acid metabolism and immune dysregulation.^[^ ^16^ ^]^ Given the significant involvement of gut microbiota in PCOS, strategies aimed at modulating the gut microbiome may provide novel therapeutic approaches.^[^ ^19^ ^]^ Inulin, a naturally soluble dietary fiber and prebiotic, is fermented by gut bacteria in the colon to produce short‐chain fatty acids (SCFAs) and reshape microbial composition, both of which directly and indirectly benefit host health.^[^ ^20^, ^21^, ^22^ ^]^

Dietary interventions, as part of lifestyle management, are recommended in clinical guidelines for PCOS. Notably, low dietary fiber intake has been reported in women with PCOS.^[^ ^23^, ^24^ ^]^ Dietary inulin supplementation has demonstrated protective effects against various metabolic disorders.^[^ ^20^ ^]^ For instance, a study showed that 42 days of inulin supplementation improved chronic inflammation and insulin resistance while increasing the abundance of *Bifidobacterium faecale* in the intestines of overweight and obese women.^[^ ^25^ ^]^ Similarly, in animal models, inulin intervention alleviated symptoms across different stages of T2D,^[^ ^26^ ^]^ and promoted the growth of *Bifidobacterium adolescentis* and the concentrations of acetic acid (AA) and propionic acid (PA) in the intestine.^[^ ^27^ ^]^ A randomized controlled trial further revealed that patients with PCOS receiving 10 g of daily inulin supplementation for 12 weeks experienced improvements in body mass, hyperandrogenism, insulin resistance, and inflammatory status.^[^ ^28^, ^29^ ^]^ However, this study did not assess changes in gut microbiota. Given its affordability and efficacy as a prebiotic, it is highly worthwhile to explore whether inulin can ameliorate PCOS by modulating the gut microbiota.

In the current study, we investigated the effects of inulin on gut microbial composition and function in a PCOS‐like mouse model and analyzed the potential ecological interactions within the gut microbiota. We also established a clinical cohort of inulin intervention in patients with PCOS to evaluate its clinical efficacy and characterize the gut microbiota changes induced by inulin. To further elucidate the mechanisms by which gut microbiota contribute to PCOS progression, we conducted fecal microbiota transplantation (FMT) experiments. Our findings provide valuable insights into the critical role and underlying mechanisms of gut microbiota in ameliorating PCOS.

## 2 Results

### 2.1 Inulin Alleviates Metabolic and Ovarian Dysfunction in PCOS Mice

**Figure 1:**
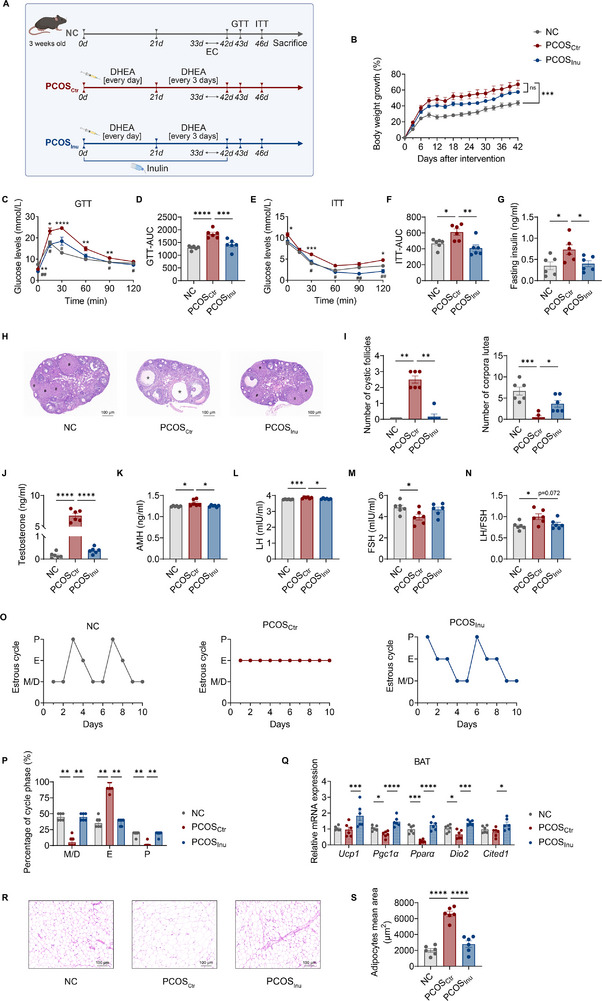
Inulin alleviates metabolic disorders and ovarian dysfunction in PCOS mice. A) Schematic diagram of the PCOS‐like mouse model with or without inulin treatment. NC, normal control mice with a chow diet and normal drinking water; PCOS_Ctr_, mice injected with DHEA and fed with a high‐fat diet (HFD) and normal drinking water; PCOS_Inu_, mice injected with DHEA, fed with HFD, and treated with inulin in drinking water; EC, estrous cycles; GTT, intraperitoneal glucose tolerance test; ITT, intraperitoneal insulin tolerance test. B) Percentage growth in body weight during the experimentation. C and D) Blood glucose levels of NC, PCOS_Ctr_, and PCOS_Inu_ mice in GTT (C) and area under the curve (AUC) of GTT (D). E and F) Blood glucose levels of NC, PCOS_Ctr_, and PCOS_Inu_ mice in ITT (E) and AUC of ITT (F). G) Fasting insulin levels of NC, PCOS_Ctr_, and PCOS_Inu_ mice. H) Representative H&E‐stained histological sections of ovaries (5×, scale bar = 100 µm) from NC, PCOS_Ctr_, and PCOS_Inu_ mice. ＊indicates cystic follicle; # indicates corpora luteum. I) Number of cystic follicles and corpora luteum. J) Serum testosterone levels of NC, PCOS_Ctr_, and PCOS_Inu_ mice. K) Serum antimullerian hormone (AMH) levels of NC, PCOS_Ctr_, and PCOS_Inu_ mice. L‐N) Serum luteinizing hormone (LH) (L) and follicle‐stimulating hormone (FSH) (M) levels and LH‐to‐FSH ratios (LH/FSH) (N) of NC, PCOS_Ctr_, and PCOS_Inu_ mice. O) Representative estrous cycles of NC, PCOS_Ctr_, and PCOS_Inu_ mice. P, proestrus; E, estrus; M, metestrus; D, diestrus. P) Quantitative analysis of each phase in estrous cycles. Q) RT‐qPCR analysis of mRNA expression levels of *Ucp1, Pgc1a, Pparα, Dio2*, and *Cited1* in the brown adipose tissue (BAT) from NC, PCOS_Ctr_, and PCOS_Inu_ mice. R) Representative H&E‐stained histological sections of peri‐ovarian adipose tissue (20×, scale bar = 100 µm) from NC, PCOS_Ctr_, and PCOS_Inu_ mice. S) Peri‐ovarian adipocyte mean area distribution. The data are shown as the mean ± standard error of the mean (SEM) and statistical significance was analyzed by one‐way ANOVA with Tukey's multiple comparisons test (*n* = 6 mice per group). For (C) and (E), * indicates NC versus PCOS_Ctr_; # indicates PCOS_Ctr_ versus PCOS_Inu_. *p < 0.05, **p < 0.01, ***p < 0.001, and ****p < 0.0001; #*p* < 0.05 and ##*p* < 0.01.

To investigate the effects of inulin on metabolism and ovarian function in PCOS, we induced PCOS‐like phenotypes in 3‐week‐old C57BL/6 female mice using dehydroepiandrosterone (DHEA)^[^ ^16^ ^]^ or in combination with a high‐fat diet (HFD)^[^ ^30^ ^]^ (Figure S1A, Supporting Information). We observed that only PCOS‐like mouse model induced by the combination of DHEA and HFD exhibited significant metabolic abnormalities, including weight gain (Figure S1B, Supporting Information), impaired glucose tolerance (Figure S1C,D, Supporting Information), and insulin resistance (Figure S1E,F, Supporting Information). In the absence of HFD, DHEA treatment alone induced abnormal ovarian morphology (Figure S1G,H, Supporting Information), disrupted estrous cycle (Figure S1I, Supporting Information), and hyperandrogenemia (Figure S1J, Supporting Information) in mice. Given the high prevalence of metabolic dysfunction in clinical PCOS,^[^ ^4^ ^]^ the PCOS‐like mouse model established by the combination of DHEA and HFD was selected for subsequent evaluations of the comprehensive effects of inulin on PCOS. Mice without inulin treatment were designated as PCOS_Ctr,_ while those treated with inulin in drinking water were designated as PCOS_Inu_. A group of mice without induction of PCOS was designated as the normal control (NC) (**Figure** **1**A). To determine the optimal inulin dosage, we tested three concentrations: low (2% w/v), medium (4% w/v), and high (8% w/v) inulin in drinking water administered to PCOS mice (Figure S2A, Supporting Information). The low dosage did not significantly improve glucose levels in PCOS mice in the glucose tolerance test (GTT, Figure S2B,C, Supporting Information) and insulin tolerance test (ITT, Figure S2D,E, Supporting Information). In contrast, both the medium and high dosages significantly improved glucose metabolism in PCOS mice, with similar effects observed between the two dosages (Figure S2B–E, Supporting Information). Following the principle of using the minimum effective dosage, 4% (w/v) inulin was chosen for subsequent experiments.

Compared with the NC mice, PCOS_Ctr_ mice showed a significant increase in body weight (Figure 1B). Although inulin treatment reduced body weight in PCOS_Inu_ mice compared to PCOS_Ctr_ mice, the difference was not statistically significant (Figure 1B). Inulin treatment significantly improved glucose tolerance in PCOS_Inu_ mice compared to non‐treated PCOS_Ctr_ mice (Figure 1C,D). Similarly, inulin treatment improved insulin sensitivity assessed by ITT (Figure 1E,F). Moreover, inulin treatment lowered the fasting insulin levels (Figure 1G). Next, we assessed ovarian histopathology in the three groups. PCOS_Ctr_ mice showed an increased number of cystic follicles and a decreased number of corpora lutea, whereas the ovaries of NC and PCOS_Inu_ mice exhibited follicles at different developmental stages and a normal number of corpora lutea (Figure 1H,I). Importantly, inulin treatment restored serum testosterone levels to normal (Figure 1J). Moreover, inulin treatment reduced the levels of antimullerian hormone (AMH, Figure 1K) and luteinizing hormone (LH, Figure 1L). Although follicle‐stimulating hormone (FSH) levels were not significantly affected by inulin (Figure 1M), the LH‐to‐FSH ratios (Figure 1N) were significantly lower in PCOS_Inu_ mice compared to PCOS_Ctr_ mice. To evaluate ovarian function, we performed estrous cycle testing and found that PCOS_Ctr_ mice exhibited disrupted estrous cycles, whereas NC and PCOS_Inu_ mice displayed regular estrous cycles (Figure 1O,P).

It was reported that brown adipose tissue (BAT), a key thermogenic organ, can effectively rescue DHEA‐induced PCOS phenotypes in rats^[^ ^31^ ^]^ and BAT activation is considered a potential therapeutic target for PCOS.^[^ ^32^ ^]^ To investigate the role of BAT in our study, we analyzed thermogenic markers in BAT from the mice using qPCR. The results showed that relative expression levels of *Pgc1α, Pparα*, and *Dio2* were significantly lower in PCOS_Ctr_ mice as compared to the NC mice. In contrast, inulin treatment significantly upregulated the expression of *Ucp1, Pgc1α, Pparα, Dio2*, and *Cited1* (Figure 1Q). Furthermore, PCOS_Ctr_ mice exhibited lipid accumulation, as evidenced by enlarged lipid droplets in peri‐ovarian adipose tissue, while inulin treatment effectively reversed this phenotype (Figure 1R,S). Taken together, our findings indicate that inulin treatment significantly improves metabolic dysregulation and ovarian function in PCOS mice.

### 2.2 Inulin Significantly Ameliorates Gut Dysbiosis in PCOS Mice by Restoring Microbial Structure and Function

**Figure 2:**
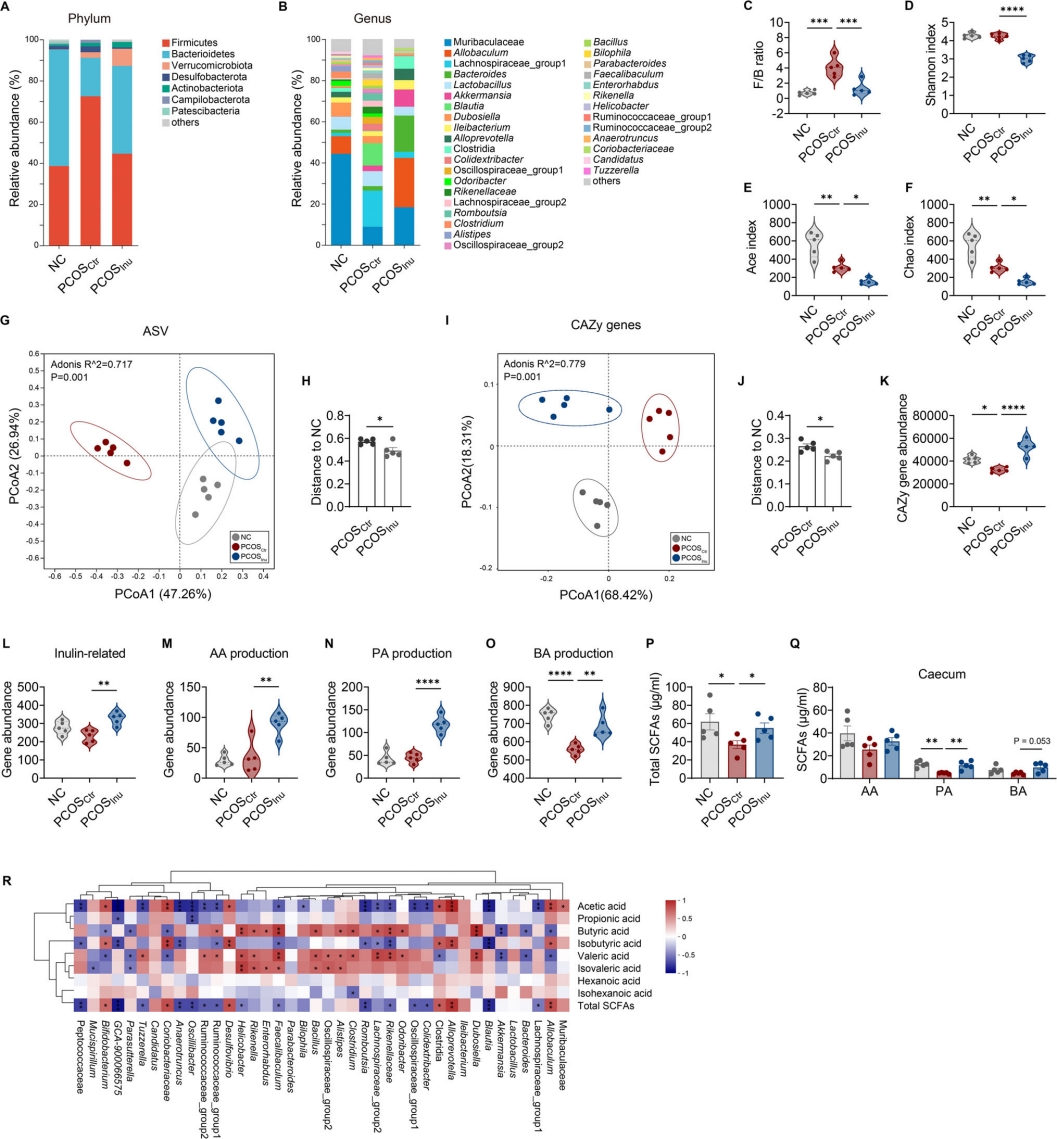
Inulin increases SCFAs‐producing bacteria in the gut of PCOS mice. A and B) Distribution of relative abundance of microbial taxa at phylum (A) and genus (B) levels in NC, PCOS_Ctr_, and PCOS_Inu_ mice. Phyla or genera with less than 1% relative abundance in the sample are classified as others. C) Ratios of Firmicutes to Bacterioidetes (F/B) in NC, PCOS_Ctr_, and PCOS_Inu_ mice. D‐F) The gut microbial community diversity (D) and richness (E and F) of NC, PCOS_Ctr_, and PCOS_Inu_ mice. G and H) Overall structure of gut microbiota in NC, PCOS_Ctr_, and PCOS_Inu_ mice. Principal coordinate analysis (PCoA) based on the weighted UniFrac distance of amplicon sequence variants (ASVs) and between‐group differences determined by Adonis analysis (G). The overall gut microbial structure of PCOS_Inu_ mice is more similar to NC mice (H). I and J) PCoA based on the Bray‐Curtis distance of carbohydrate‐active enzyme (CAZy) family genes in NC, PCOS_Ctr_, and PCOS_Inu_ mice (I). The CAZy family genes of PCOS_Inu_ mice are more similar to NC mice (J). K) The abundance of CAZy family genes in NC, PCOS_Ctr_, and PCOS_Inu_ mice. L) The abundance of CAZy genes (GH32 and GH91) involved in inulin metabolism in NC, PCOS_Ctr_, and PCOS_Inu_ mice. M‐O) Alterations in the abundance of genes encoding key enzymes in the production pathways for (M) acetic acid (AA), (N) propionic acid (PA), and (O) butyric acid (BA) in NC, PCOS_Ctr_, and PCOS_Inu_ mice. AA production: formate‐tetrahydrofolate ligase; PA production: propionyl‐CoA:succinate‐CoA transferase and propionate CoA‐transferase; BA production: represented by the total abundances of genes encoding the following enzymes: 4Hbt, butyryl–coenzyme A (butyryl‐CoA): 4‐hydroxybutyrate CoA transferase; Ato, butyryl‐CoA: acetoacetate CoA transferase; Buk, butyrate kinase; But, butyryl‐CoA: acetate CoA transferase. P and Q) The concentration of total (P) and three major short‐chain fatty acids (SCFAs) including AA, PA, and BA (Q) in the caecum of NC, PCOS_Ctr_, and PCOS_Inu_ mice. R) Heatmap of the Spearman's correlation between the top 40 most abundant bacteria genera and SCFAs in mice. Red squares represent positive correlations, while blue squares represent negative correlations. P‐values less than 0.05 are marked with asterisks. *p < 0.05, **p < 0.01, and ***p < 0.001. For (C)‐(F) and (K)‐(O), data are shown as violin plots with the median, interquartile ranges (IQRs), and min/max values; for (H) and (J), data are presented as the mean ± SEM. Statistical significance was analyzed by one‐way ANOVA with Tukey's multiple comparisons test (*n* = 5 mice per group). *p < 0.05, **p < 0.01, ***p < 0.001 and ****p < 0.0001. [Correction added on 21 April 2025, after first online publication: Figure 2 is updated in this version.]

Since inulin, as a common dietary fiber, is known to influence gut microbiota composition,^[^ ^20^ ^]^ we next performed high‐throughput 16S rRNA gene sequencing to profile gut microbial taxonomic signatures in each group of mice. At the phylum level, PCOS_Ctr_ mice exhibited a higher abundance of Firmicutes and a lower abundance of Bacterioidetes (**Figure** **2**A), along with a significantly increased Firmicutes‐to‐Bacterioidetes (F/B) ratio (Figure 2C). Inulin treatment reversed these alterations. At the genus level, the dominant genera differed significantly among the three groups (Figure 2B). Compared with NC mice, PCOS_Ctr_ mice exhibited gut dysbiosis, characterized by a decreased abundance of Muribaculaceae and *Allobaculum*, and an increased abundance of Lachnospiraceae (Figure 2B; Figure S3A,B, Supporting Information). It is noteworthy that inulin administration increased the abundance of Muribaculaceae (Figure 2B) and five genera associated with the bacteria known to produce SCFAs, including *Allobaculum, Bacteroides, Akkermansia, Alloprevotella*, and *Bifidobacterium*, as identified by Linear discriminant analysis effect size (LEfSe) (Figure S3A,B, Supporting Information).

When assessing microbial community diversity, we found no significant difference in alpha diversity between NC and PCOS_Ctr_ mice by the Shannon index. However, inulin treatment resulted in a significant decrease in the Shannon index in PCOS_Inu_ mice (Figure 2D). Interestingly, analysis using the Ace and Chao indices revealed that both PCOS_Ctr_ and PCOS_Inu_ mice had significantly lower microbial community richness compared with NC mice, with PCOS_Inu_ mice showing the lowest richness (Figure 2E,F). Based on the principal coordinate analysis (PCoA) plot of amplicon sequence variants (ASVs), there were significant differences in overall gut microbiota structure among the three groups (P = 0.001; R‐squared = 0.717) (Figure 2G). Notably, we observed shorter distances between samples from the NC and PCOS_Inu_ groups, as measured by weighted UniFrac distances (Figure 2H) or Jaccard distances (Figure S3C, Supporting Information), indicating that the gut microbiota structure in PCOS_Inu_ mice was more alike to that in NC mice.

The microbial functional profiling was further analyzed by metagenomic sequencing. Carbohydrate‐active enzyme (CAZy) family genes,^[^ ^33^ ^]^ which encode carbohydrate‐active enzymes that degrade complex polysaccharides like dietary fiber into fermentable substrates, are essential for SCFAs production. Principal coordinate analysis (PCoA) based on the Bray‐Curtis distances of CAZy genes indicated significant differences among groups by Adonis (P = 0.001; R‐squared = 0.779) (Figure 2I). In addition, shorter distances were observed between samples from the NC and PCOS_Inu_ groups, based on the Bray‐Curtis distances or Jaccard distances of CAZy genes (Figure 2J; Figure S3D, Supporting Information), and Kyoto Encyclopedia of Genes and Genomes (KEGG) orthologs (KOs) (Figure S3E,F, Supporting Information). These results indicated that the gut microbial function of PCOS_Inu_ mice closely resembles that of NC mice. Moreover, the total abundance of CAZy genes was significantly lower in the PCOS_Ctr_ mice compared with NC mice, but inulin treatment significantly increased CAZy gene abundance in the gut microbiota (Figure 2K). Notably, CAZy genes involved in inulin metabolism were significantly enriched in PCOS_Inu_ mice (Figure 2L). Focusing on SCFAs metabolism, we annotated key enzymes involved in the production pathways of AA, PA, and butyric acid (BA), as previously described.^[^ ^34^, ^35^ ^]^ The gut microbiota in PCOS_Ctr_ showed significantly decreased gene abundance related to BA production (Figure 2O), particularly the gene encoding butyryl–coenzyme A (butyryl‐CoA): 4‐hydroxybutyrate CoA transferase (4Hbt) (Figure S3G, Supporting Information). The production of AA, PA, and BA was promoted by inulin supplementation (Figure 2M–O). In addition, discriminatory genera enriched by inulin exhibited significantly positive correlations with AA and PA production (Figure S4, Supporting Information). Beyond genomic data, we measured SCFAs in cecal contents. Compared with NC mice, PCOS_Ctr_ mice had significantly lower total SCFAs (Figure 2P) and PA (Figure 2Q). In contrast, inulin enhanced total SCFAs in the cecum of PCOS_Inu_ mice (Figure 2P), with significant increases in PA and BA (Figure 2Q). Spearman's correlation analysis revealed that inulin‐enriched bacteria, such as *Bifidobacterium, Alloprevotella, Allobaculum*, and Muribaculaceae, were significantly positively correlated with AA and total SCFAs (Figure 2R).

In addition, we performed an alignment analysis based on a comprehensive antibiotic resistance database (CARD)^[^ ^36^ ^]^ and a virulence factors database (VFDB).^[^ ^37^ ^]^ From volcano plots, PCOS_Ctr_ mice showed more up‐regulated antibiotic resistance genes (ARGs) and virulence factor genes (VFGs) than NC mice, whereas inulin down‐regulated ARGs and VFGs in PCOS_Inu_ mice (Figure S5, Supporting Information). Using LEfSe for level‐three pathways in KEGG database, we found that numerous crucial metabolic pathways associated with carbohydrates, amino acids, and nucleic acids were mainly enriched in NC and PCOS_Inu_ mice, while pathways related to infection were enriched in PCOS_Ctr_ mice (Figure S6, Supporting Information). Overall, inulin altered the comprehensive function of the gut microbiota in PCOS mice, featured by significantly enhancing microbial capacity for carbohydrate metabolism and facilitating SCFAs production.

**Figure 3:**
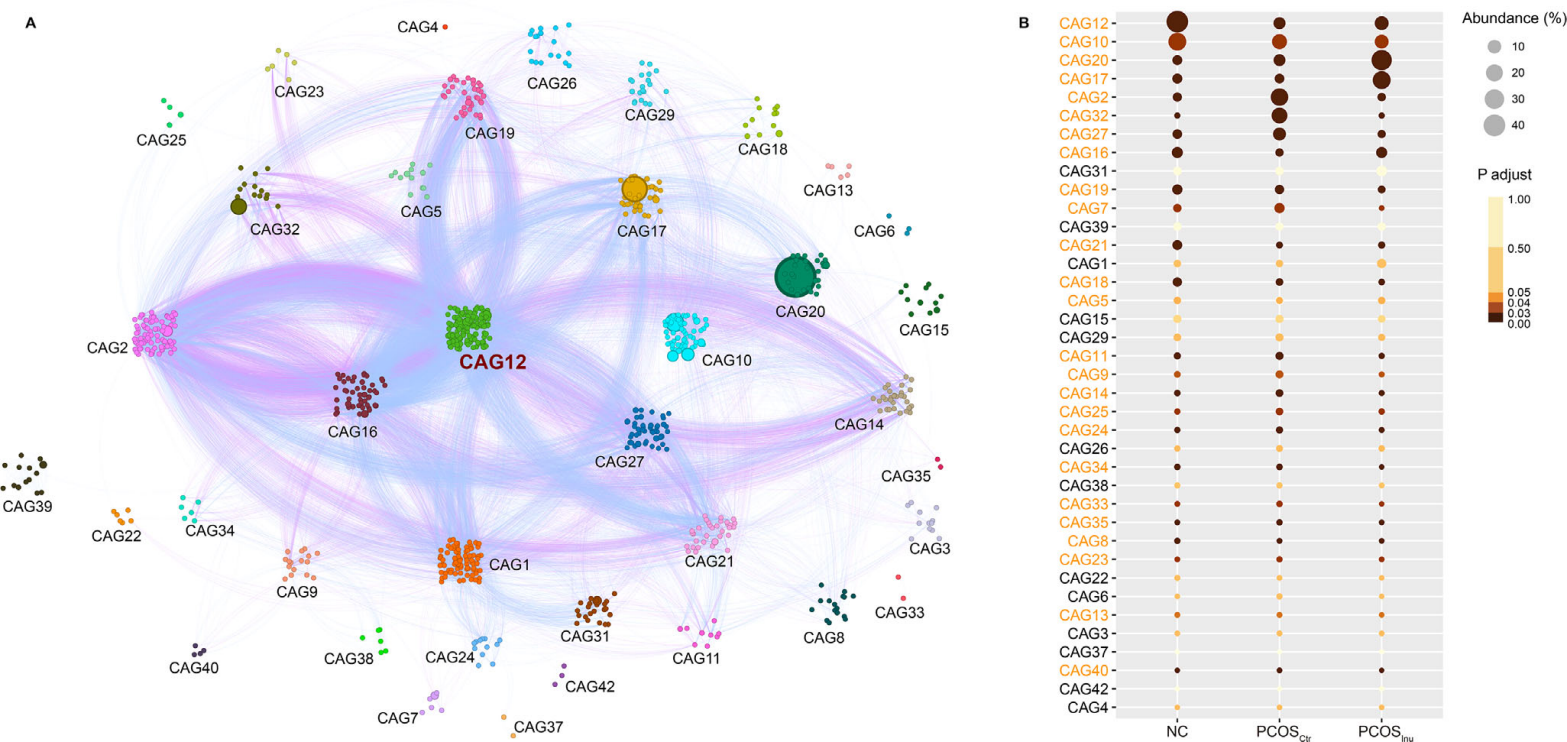
Inulin modifies microbial co‐abundant groups (CAGs) in PCOS mice. A) The interaction between different CAGs by microbial co‐abundance network. The node size reflects the mean abundance of species, with larger nodes corresponding to higher abundance. The lines connecting the nodes reflect correlations (pink represents negative correlation, blue represents positive correlation), with the line width indicating the strength of the correlation. B) Changes in the abundance of CAGs in NC, PCOS_Ctr_, and PCOS_Inu_ mice. The sizes and colors of circles indicate the relative abundance and the adjusted P value of CAGs, respectively. The CAG number highlighted in orange indicates significant differences analyzed by the Kruskal‐Wallis test (*n* = 5 per group).

To elucidate the potential interactions in the symbiotic ecosystem of gut microbiota, we constructed a microbial co‐abundance network among species shared at least 80% of all samples. The SparCC correlation analysis and PERMANOVA were used to identify 42 co‐abundance groups (CAGs), visualized as a topological lotus diagram (**Figure** **3**A). We found that CAG12 was the group with the highest abundance and diversity of species, and it closely interacted with other CAGs, suggesting that CAG12 was an important core gut microbial community in the mice. Interestingly, several *Bifidobacterium* species were found within CAG12, and many species belonging to the Muribaculaceae family were also present in CAG12. Additionally, we observed a highly significant positive correlation (blue line) between CAG12 and CAG16. High abundances of Muribaculaceae and *Bifidobacterium* also existed in CAG16. Moreover, CAG16 contained high abundances of beneficial bacteria such as *Parabacteroides distasonis* ^[^ ^38^ ^]^ and *Bacteroides thetaiotaomicron*.^[^ ^39^ ^]^ Meanwhile, CAG12 exhibited distinctly negative correlations (pink line) with CAG2 and CAG32, both enriched with species belonging to the Lachnospiraceae family. Importantly, compared to NC mice, the abundances of CAG12 and CAG16 were significantly decreased in PCOS_Ctr_ mice. Conversely, CAG2 and CAG32, which were negatively correlated with CAG12, were significantly enriched in PCOS_Ctr_ mice. However, inulin significantly promoted an increase in CAG12 and CAG16, accompanied by a decrease in CAG2 and CAG32 in PCOS_Inu_ mice. Additionally, CAG20 (represented by Muribaculaceae) and CAG17 (represented by *Bacteroides acidifaciens*) were significantly enriched in PCOS_Inu_ mice (Figure 3B). Collectively, inulin modulated the composition and interactions of microbiota within the gut ecosystem in PCOS mice. The complete matrix of the CAGs analysis is provided in Supplementary Data1.

### 2.3 Inulin Strengthens Intestinal Barrier and Relieves Inflammation in PCOS Mice

**Figure 4:**
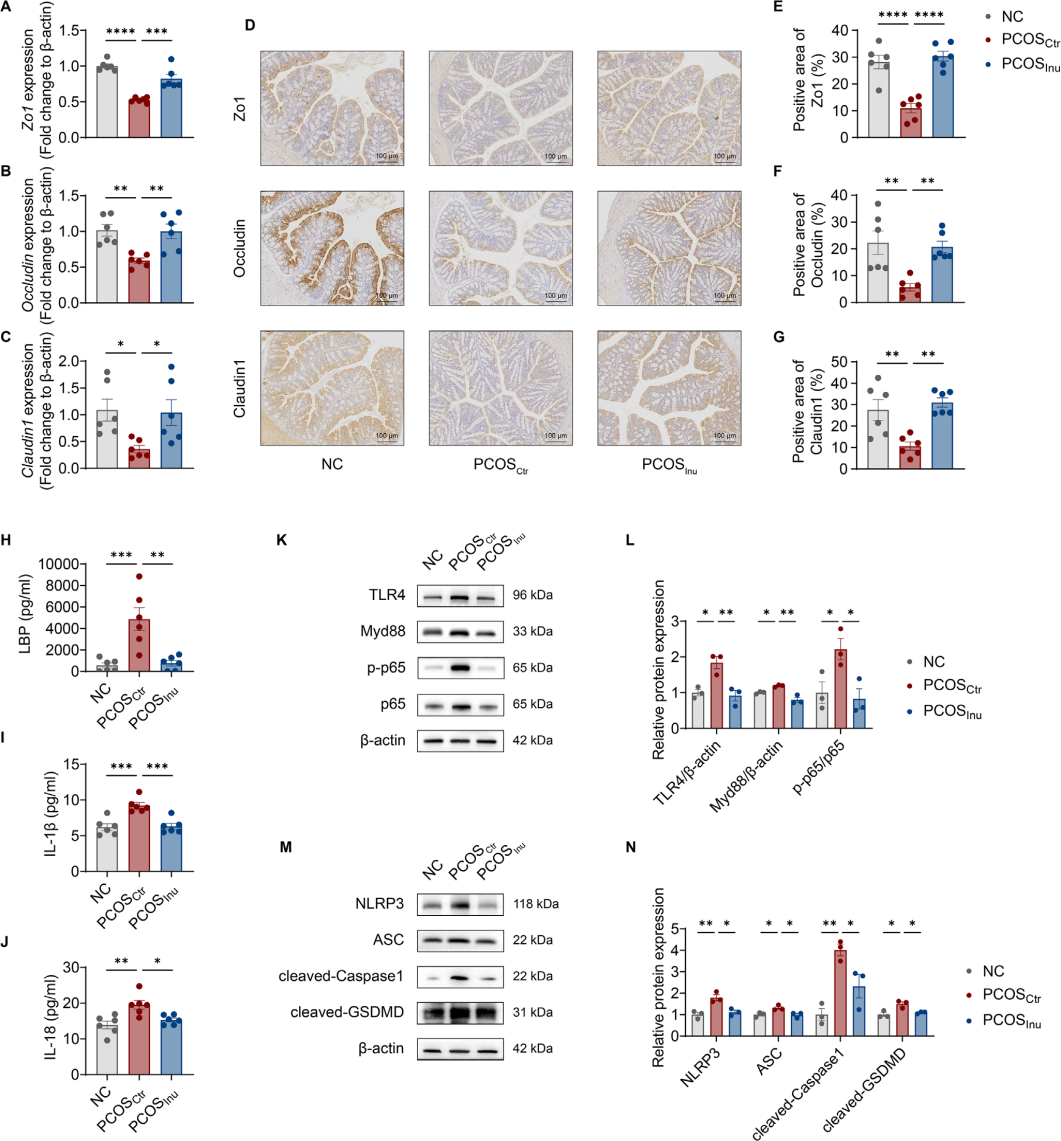
Inulin ameliorates impaired intestinal barrier and ovarian inflammation in PCOS mice. A‐C) RT‐qPCR analysis of mRNA expression levels of *Zo1, Occludin*, and *Claudin1* in the colon from NC, PCOS_Ctr_, and PCOS_Inu_ mice. D‐G) Immunohistochemical staining and analysis of Zo1, Occludin, and Claudin1 in the colon from NC, PCOS_Ctr_, and PCOS_Inu_ mice (10×, scale bar = 100 µm). H‐J) Serum levels of LBP (H), IL‐1β (I), and IL‐18 (J) in NC, PCOS_Ctr_, and PCOS_Inu_ mice. K‐N) Western blotting bands presenting protein expression levels of TLR4, Myd88, p‐NF‐κB (K) and NLRP3, ASC, cleaved‐Caspase1, cleaved‐GSDMD (M) in the ovary from NC, PCOS_Ctr_, and PCOS_Inu_ mice. Relative protein expression levels were determined via quantification of band intensities normalized by β‐actin (L and N). The data are shown as the mean ± SEM and statistical significance was analyzed by one‐way ANOVA with Tukey's multiple comparisons test. For (A)‐(G) and (L)‐(N), *n* = 6 mice per group; for (H)‐(K), *n* = 3 mice per group. *p < 0.05, **p < 0.01, ***p < 0.001, and ****p < 0.0001.

The levels of SCFAs in the intestine have been strongly associated with the integrity of the intestinal mucosal barrier and susceptibility to inflammation.^[^ ^40^ ^]^ Therefore, we utilized qPCR (**Figure** **4**A–C) and immunohistochemistry (Figure 4D–G) to examine the transcription and expression of the genes encoding for tight‐junction proteins, including Zo1, Occludin, and Claudin1, from the colonic tissues of mice in the study. There was a significant decrease in the expression levels of tight‐junction genes and proteins in PCOS_Ctr_ mice, which were upregulated by inulin treatment (Figure 4A–G). These data indicate that inulin treatment could markedly inhibit the integrity disruption of the intestinal barrier in PCOS mice.

Given the crucial role of intestinal barrier integrity in preventing the translocation of bacterial lipopolysaccharide (LPS) into systemic circulation,^[^ ^41^ ^]^ we measured serum LPS‐binding protein (LBP), reflecting circulating LPS level. Consistent with the reduced expression of tight‐junction‐related genes and proteins observed in PCOS_Ctr_ mice, serum LBP levels were significantly elevated in PCOS_Ctr_ mice compared to both NC and PCOS_Inu_ mice (Figure 4H). Serum levels of pro‐inflammatory cytokines interleukin‐1 beta (IL‐1β) and interleukin‐18 (IL‐18), were also significantly elevated in PCOS_Ctr_ mice compared to both NC and PCOS_Inu_ mice (Figure 4I,J). To investigate the interplay between inulin and inflammation in PCOS, we administered LPS intraperitoneally to inulin‐treated PCOS mice, designated as PCOS_Inu+LPS._ (Figure S7A, Supporting Information). Although LPS exposure did not result in significant growth in body weight (Figure S7B, Supporting Information), it induced dysglycemia (Figure S7C,D, Supporting Information), insulin resistance (Figure S7E,F, Supporting Information), increased cystic follicles and decreased corpora luteum (Figure S7G,H, Supporting Information), and disrupted estrous cycle (Figure S7I, Supporting Information) in PCOS_Inu+LPS_ mice. Notably, PCOS_Inu+LPS_ mice presented chronic low‐grade inflammation phenotype, similar to PCOS_Ctr_ mice, as evidenced by elevated serum levels of LBP, IL‐1β, and IL‐18 (Figure S7J–L, Supporting Information) and upregulated ovarian mRNA expression levels of *Lbp, Il1b*, and *Il18* (Figure S7M, Supporting Information). These results demonstrate that LPS administration reverses inulin‐mediated amelioration of PCOS pathology, highlighting the significance of intestinal barrier integrity in suppressing inflammation.

Inflammation has been considered as one of the important factors in PCOS pathogenesis,^[^ ^42^, ^43^ ^]^ however, it is not clear if innate immunity is involved in the inflammatory process in PCOS. The LEfSe analysis revealed significant enrichment of the NOD‐like receptor (NLR) signaling pathway^[^ ^44^ ^]^ in PCOS_Ctr_ mice (Figure S6, Supporting Information). Thus, we assessed ovarian mRNA expression levels of *Nlrp3*, *Nlrp1*, and *Nlrc4*, among which the expression of *Nlrp3* mRNA levels was upregulated significantly in both PCOS_Ctr_ and PCOS_Inu+LPS_ mice (Figure S7N, Supporting Information). Next, we assessed the upstream signaling molecules, including toll‐like receptor 4 (TLR4), myeloid differential protein 88 (Myd88), phosphorylated‐nuclear factor (NF)‐κB (p‐p65), as well as downstream pyroptosis‐related proteins, including NLR pyrin domain containing protein 3 (NLRP3), apoptosis‐associated speck‐like protein containing CARD (ASC), cysteinyl aspartate specific proteinase 1 (Caspase1), and gasdermin D (GSDMD) in ovarian tissue by western blot. We found that the expression levels of TLR4, Myd88, and p‐p65/p65 in the ovarian tissue of PCOS_Ctr_ mice were significantly increased compared to both NC and PCOS_Inu_ mice (Figure 4K,L). In addition, compared with NC mice, PCOS_Ctr_ mice showed increased expression levels of inflammasome NLRP3/ASC/cleaved‐Caspase‐1 and cleaved‐GSDMD, suggesting the activation of the pyroptosis pathway. However, inulin treatment significantly mitigated activation of the NLRP3 inflammasome and suppressed pyroptosis in the ovarian tissue of PCOS_Inu_ mice (Figure 4M,N). Our results suggest that the inulin treatment improves the integrity of the intestinal barrier and suppresses ovarian inflammation in PCOS mice.

### 2.4 Improvement of Clinical Parameters and Gut Microbiota in Patients with PCOS after Administration of Inulin

**Figure 5:**
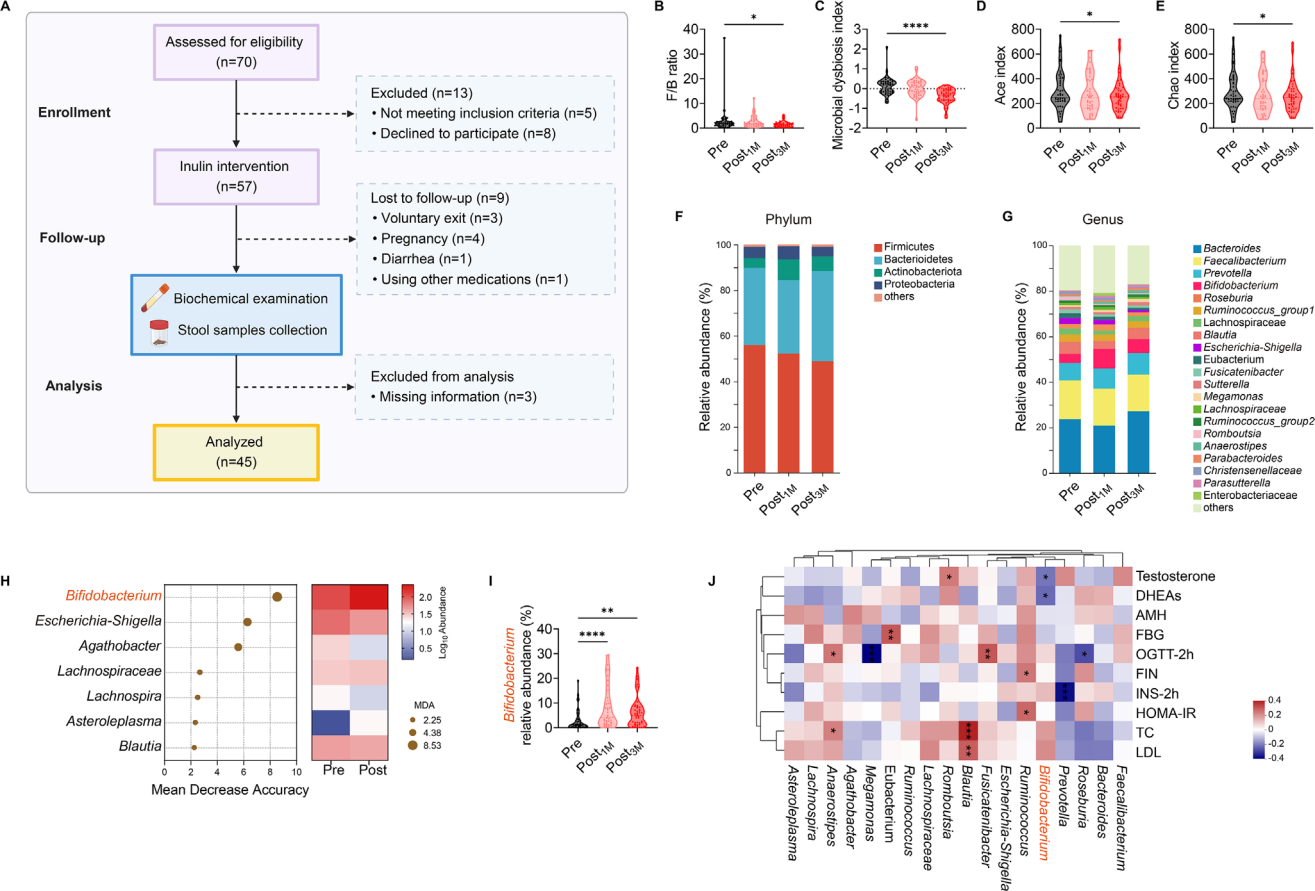
Alteration of gut microbiota in patients with PCOS after inulin intervention. A) Flow chart of the study design. B) Ratios of Firmicutes to Bacterioidetes (F/B) among patients with PCOS pre‐inulin (Pre), 1 month post‐inulin (Post_1_ _M_), and 3 months post‐inulin (Post_3_ _M_) intervention. C) The microbial dysbiosis index of Pre, Post_1_ _M_, and Post_3_ _M_ groups. D and E) The alpha‐diversity of gut microbiota in Pre, Post_1_ _M_, and Post_3_ _M_ groups. F and G) Distribution of relative abundance of microbial taxa at phylum (F) and genus (G) levels in Pre, Post_1_ _M_, and Post_3_ _M_ groups. Phyla or genera with less than 1% relative abundance in the sample are classified as others. H) Classification performance of the 7 most discriminant genera by a random forest model. I) Comparison of relative abundance of *Bifidobacterium* in the gut of patients with PCOS between Pre and Post groups. J) Heatmap of the Spearman's correlation between key bacteria genera and clinical parameters in patients with PCOS. Red squares represent positive correlations, while blue squares represent negative correlations. P‐values less than 0.05 are marked with asterisks. *p < 0.05, **p < 0.01, and ***p < 0.001. For (B)‐(E) and (I), data are shown as violin plots with the median, interquartile ranges (IQR), and min/max values; two‐tailed Wilcoxon matched‐pairs test was used to analyze differences between the Pre and Post_1_ _M_ groups or the Pre and Post_3_ _M_ groups. The sample size: *n *= 45 patients per group. *p < 0.05, **p < 0.01, ***p < 0.001, and ****p < 0.0001.

To further investigate whether inulin has the equally health benefits for patients with PCOS, we performed a prospective self‐controlled clinical trial. Women diagnosed with PCOS, based on the 2003 Rotterdam criteria,^[^ ^45^ ^]^ were enrolled in this study and received 10 g of inulin daily for three months. A total of 45 subjects were included in the study under strict inclusion and exclusion criteria, with a mean age of 29.53 ± 3.06 (**Figure** **5**A). As shown in **Table** **1**, the subjects had significantly lower sex hormones, including testosterone, dehydroepiandrosterone sulfate (DHEAs), and AMH at the end of the study. Moreover, the subjects had improved glucose metabolism at the end of the study, including lowered fasting blood glucose (FBG), fasting insulin (FIN), blood insulin level at the 2‐hour time point of the oral glucose tolerance test (INS‐2 h), and homeostatic model of assessment of insulin resistance (HOMA‐IR). In addition, there was a significant decline in body mass index (BMI) and total cholesterol (TC) levels as well as a decreased trend in low‐density lipoprotein (LDL). Overall, the metabolic status of study subjects improved significantly after inulin intervention.

**Table 1:** Clinical parameters of patients with PCOS before and after inulin intervention.

Clinical Parameters	Baseline	End of Study	*T*	*Z*	*P*
Testosterone (ng/mL)	0.34 (0.28, 0.42)	0.29 (0.25, 0.37)	–	−2.739	**0.006** ^a^
DHEAs (umol/L)	7.97 ± 3.40	6.74 ± 2.74	4.512	–	**0.000** ^b^
AMH (ng/mL)	8.56 (5.82, 11.86)	7.01 (5.22, 10.24)	–	−2.878	**0.004** ^a^
FBG (mmol/L)	4.80 (4.50, 5.10)	4.60 (4.50, 4.80)	–	−3.356	**0.001** ^a^
OGTT‐2 h (mmol/L)	5.80 (5.10, 6.50)	5.50 (4.81, 6.20)	–	−1.734	0.083^a^
FIN (μIU/mL)	9.45 (5.97, 12.15)	7.16 (4.87, 9.84)	–	−3.968	**0.000** ^a^
INS‐2 h (μIU/mL)	56.28 (40.70, 100.11)	42.44 (25.83, 72.75)	–	−2.778	**0.005** ^a^
HOMA‐IR	1.91 (1.24, 2.65)	1.37 (0.94, 2.03)	–	−4.058	**0.000** ^a^
HbA1c (%)	5.43 ± 0.34	5.43 ± 0.32	0.077	–	0.939^b^
TC (mmol/L)	4.74 (4.32, 5.18)	4.54 (4.03, 4.95)	–	−2.252	**0.024** ^a^
TG (mmol/L)	0.99 (0.69, 1.30)	0.86 (0.64, 1.45)	–	−0.593	0.553^a^
HDL (mmol/L)	1.46 ± 0.37	1.43 ± 0.29	0.726	–	0.471^b^
LDL (mmol/L)	3.00 ± 0.79	2.83 ± 0.80	1.979	–	0.054^b^
BMI (kg/m^2^)	22.76 (20.08, 26.25)	22.11 (19.56, 24.77)	–	−5.525	**0.000** ^a^

Data are shown as the mean ± standard deviation (SD) or the median with interquartile range (IQR). The p values are calculated based on a. Wilcoxon signed rank test or *b*. Paired‐samples Student's t test, with the bold font indicating a *p*‐value less than 0.05. DHEAs, dehydroepiandrosterone sulfate; AMH, antimullerian hormone; FBG, fasting blood glucose; OGTT‐2 h, blood glucose detected at the two‐hour point of the oral glucose tolerance test; FIN, fasting insulin; INS‐2 h, blood insulin detected at the two‐hour point of the oral glucose tolerance test; HOMA‐IR, homeostatic model of assessment of insulin resistance; HbA1c, hemoglobin A1c; TC, total cholesterol; TG, triglyceride; HDL, high‐density lipoprotein; LDL, low‐density lipoprotein; BMI, body mass index.

It is known that gut microbiota play an important role in the host metabolism, to explore the effect of inulin, a soluble fiber, on the gut microbiota of the women with PCOS, we collected fecal samples from the study subjects at three time points, including pre‐inulin intervention (Pre), 1 month post‐inulin intervention (Post_1_ _M_) and 3 months post‐inulin intervention (Post_3_ _M_), and performed the 16S rRNA gene sequencing. Compared to the pre‐inulin intervention, we found the reduced F/B ratio in the Post_1_ _M_ and Post_3_ _M_ time points and the reduction was statistically significant at the 3‐month time point (Figure 5B,F). The microbial dysbiosis index, which was calculated based on the abundance of taxa, decreased significantly in Post_3_ _M_ samples compared with Pre samples (Figure 5C). Furthermore, the Ace and Chao indices reflecting the alpha diversity of the microbiota community were significantly lower in Post_3_ _M_ samples than in Pre samples (Figure 5D,E). However, the overall structure of gut microbiota did not differ significantly among Pre, Post_1_ _M_, and Post_3_ _M_ groups, as displayed in PCoA based on the weighted UniFrac distance of ASVs with Adonis (P = 0.197; R‐squared = 0.020) (Figure S8A, Supporting Information). Aiming at identifying the relevant differential bacteria, we further analyzed the data at different taxonomy levels. At the phylum level, both Post_1_ _M_ and Post_3_ _M_ groups showed a higher abundance of Actinobacteriota than the Pre group (Figure 5F; Figure S8C–F, Supporting Information). At the genus level, both Post_1_ _M_ and Post_3_ _M_ groups had a higher abundance of *Bifidobacterium* but a lower abundance of *Escherichia‐Shigella* compared to Pre group (Figure 5G–I; Figure S8C–F, Supporting Information). In a random forest model, a group of seven genera was selected as the key genera with the most discriminability between the Pre‐ and Post group, with AUC values of 0.687 (Figure 5H; Figure S8B, Supporting Information). *Bifidobacterium* exhibited the highest mean decrease accuracy (MDA) (Figure 5H). As *Bifidobacterium* is an essential probiotic in the human intestine,^[^ ^46^ ^]^ we investigated the association of the abundance of *Bifidobacterium* with the clinical parameters of patients with PCOS. We found a negative correlation between the abundance of *Bifidobacterium* and testosterone or DHEAs levels (Figure 5J). Our findings indicate that inulin benefits the management of PCOS and promotes the specific bifidogenesis of gut microbiota.

### 2.5 Gut Microbiota from the Donors with Inulin Intervention Improve PCOS‐like Phenotypes in Mice

**Figure 6:**
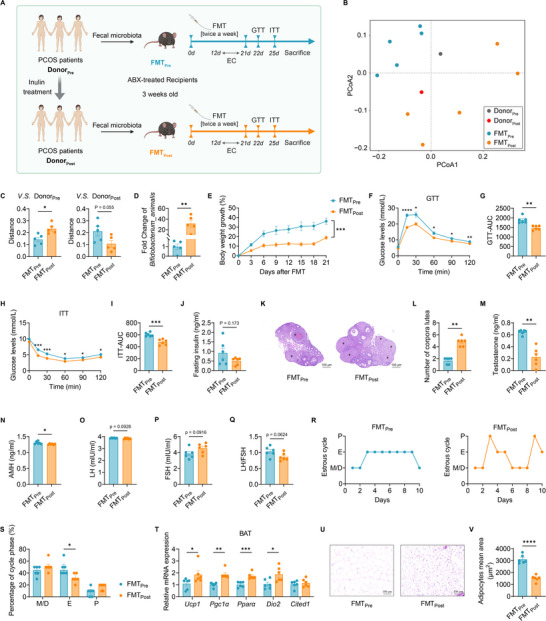
Improved metabolic outcomes in mice after the fecal microbiota transplantation of patients with PCOS post‐inulin intervention. A) Schematic diagram of the fecal microbiota transplantation (FMT) experiments. Fecal samples were collected from randomly selected three donors of patients with PCOS pre‐ and post‐inulin intervention, respectively. The mice were treated with an antibiotic cocktail prior to the FMT. FMT_Pre_, recipient mice inoculated with the pooled fecal microbiota from pre‐inulin PCOS patients (Donor_Pre_); FMT_Post_, recipient mice inoculated with the pooled fecal microbiota from post‐inulin PCOS patients (Donor_Post_). All the recipient mice were fed with HFD after FMT; EC, estrous cycles; GTT, intraperitoneal glucose tolerance test; ITT, intraperitoneal insulin tolerance test. B) PCoA based on the Bray‐Curtis distance of species in recipient mice and PCOS donors. C) The overall gut microbial structure of recipient mice is more similar to their fecal donors. D) Comparison of *Bifidobacterium animalis* based on the shallow metagenome sequencing data between FMT_Pre_ and FMT_Post_ mice. E) Percentage growth in body weight during the FMT experiment. F and G) Blood glucose levels of FMT_Pre_ and FMT_Post_ mice in GTT (F) and AUC of GTT (G). H and I) Blood glucose levels of FMT_Pre_ and FMT_Post_ mice in ITT (H) and AUC of ITT (I). J) Fasting insulin levels of FMT_Pre_ and FMT_Post_ mice. K and L) Representative H&E‐stained histological sections of ovaries (5×, scale bar = 100 µm); # indicates corpora luteum (K). Number of corpora lutea in FMT_Pre_ and FMT_Post_ mice (L). M) Serum testosterone levels of FMT_Pre_ and FMT_Post_ mice. N) Serum AMH levels of FMT_Pre_ and FMT_Post_ mice. O‐Q) Serum LH (O) and FSH (P) levels and LH‐to‐FSH ratios (Q) of FMT_Pre_ and FMT_Post_ mice. R and S) Representative estrous cycles of FMT_Pre_ and FMT_Post_ mice (R) and quantitative analysis of each phase in estrous cycles (S). P, proestrus; E, estrus; M, metestrus; D, diestrus. T) RT‐qPCR analysis of mRNA expression levels of *Ucp1, Pgc1a, Pparα, Dio2*, and *Cited1* in BAT from FMT_Pre_ and FMT_Post_ mice. U and V) Representative H&E‐stained histological sections of peri‐ovarian adipose tissue (20×, scale bar = 100 µm) from FMT_Pre_ and FMT_Post_ mice (U) and quantitative analysis of adipocyte mean area (V). The data are shown as the mean ± SEM and statistical significance was analyzed by two‐tailed Student's t‐test. For (B)‐(D), *n* = 5 mice per group; for (E)‐(V), *n* = 6 mice per group. *p < 0.05, **p < 0.01, ***p < 0.001, and ****p < 0.0001.

To prove the metabolic improvements in patients with PCOS by inulin are mediated by the gut microbiota, we designed cross‐species FMT experiments as illustrated in **Figure** **6**A, in which we transplanted human fecal microbiota to mice. To deplete the mouse endogenous gut bacteria, we treated the mice with an antibiotic cocktail for 2 weeks prior to FMT. The mice then received the pooled fecal microbiota from the donors either pre‐inulin intervention or post‐inulin intervention. The recipient mice were designated as FMT_Pre_ and FMT_Post_, respectively. We sequenced fecal samples of the recipient mice three weeks after FMT along with the donor samples by shallow metagenomic sequencing method. The overall structure of the gut microbiota of the recipient mice was more similar to that of their donors in each group, however, the microbiota structure of the two FMT groups was very different (Figure 6B,C). Interestingly, FMT_Post_ mice had a significantly higher abundance of *Bifidobacterium animalis* compared with FMT_Pre_ mice (Figure 6D).

We found that there was a significant body weight loss in FMT_Post_ mice in comparison to FMT_Pre_ mice, despite all recipient mice being fed the same HFD (Figure 6E). Blood glucose levels were also significantly lower in FMT_Post_ mice than FMT_Pre_ mice both in the GTT and ITT tests (Figure 6F–I). Compared to FMT_Pre_ mice, the serum level of fasting insulin was lower in FMT_Post_ mice, although it was not statistically significant (Figure 6J). These data demonstrate that the human gut microbiota from post‐inulin intervention contributes to maintaining normal glucose regulation and insulin sensitivity in the recipient mice. Upon histology of ovaries in the recipient mice, FMT_Post_ mice showed normal structure of ovaries with follicles at all levels and corpora lutea. The hematogenous corpus luteum was found in FMT_Pre_ mice, but the number of corpora lutea was significantly reduced compared with FMT_Post_ mice (Figure 6K,L). In addition, the serum level of testosterone was significantly lower in FMT_Post_ mice than FMT_Pre_ mice (Figure 6M). We tested serum levels of AMH (Figure 6N), LH (Figure 6O), and FSH (Figure 6P) and found that AMH and LH/FSH ratio (Figure 6Q) were decreased in FMT_Post_ mice compared to FMT_Pre_ mice. In the estrous cycle test, FMT_Post_ mice exhibited regular estrous cycles, whereas FMT_Pre_ mice spent significantly more time in the estrus stage (Figure 6R,S). These results suggest the postinulin microbiota also improves ovarian dysfunction. We further measured BAT‐associated gene expression involved in thermogenesis to assess the BAT activity. The relative expression levels of *Ucp1*, *Pgc1α*, *Pparα*, and *Dio2* were significantly higher in FMT_Post_ mice than in FMT_Pre_ mice (Figure 6T). Moreover, compared to FMT_Pre_ mice, smaller sizes of lipid droplets in peri‐ovarian adipose tissue were found in FMT_Post_ mice (Figure 6U,V), demonstrating that the post‐inulin human microbiota is conducive to enhancing BAT thermogenic activity and inhibiting lipid accumulation.

**Figure 7:**
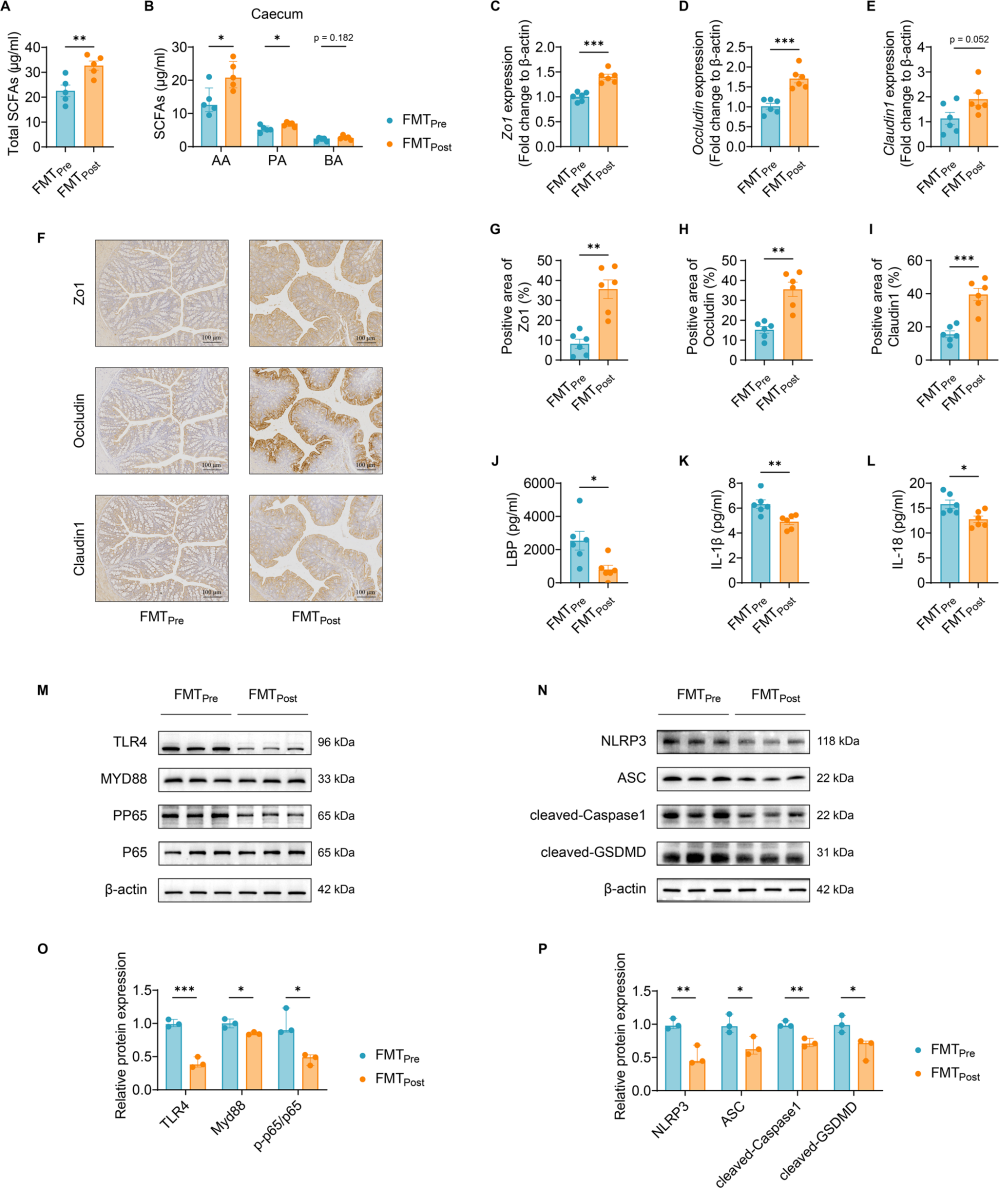
Post‐inulin microbiota increases SCFAs and enhances the intestinal barrier in mice. A and B) The concentration of total (A) and three major SCFAs (B) including AA, PA, and BA in the caecum of FMT_Pre_ and FMT_Post_ mice. C‐E) RT‐qPCR analysis of mRNA expression levels of *Zo1, Occludin*, and *Claudin1* in the colon from FMT_Pre_ and FMT_Post_ mice. F‐I) Immunohistochemical staining and analysis of Zo1, Occludin, and Claudin1 in the colon from FMT_Pre_ and FMT_Post_ mice (10×, scale bar = 100 µm). J‐L) Serum levels of LBP (J), IL‐1β (K), and IL‐18 (L) in FMT_Pre_ and FMT_Post_ mice. M‐P) Western blotting bands presenting protein expression levels of TLR4, Myd88, p‐NF‐κB (M) and NLRP3, ASC, cleaved‐Caspase1, cleaved‐GSDMD (N) in the ovary from FMT_Pre_ and FMT_Post_ mice. Relative protein expression levels were determined via quantification of band intensities normalized by β‐actin (O and P). The data are shown as the mean ± SEM and statistical significance was analyzed by two‐tailed Student's t‐test. For (A) and (B), *n* = 5 mice per group; for (C)‐(L), *n* = 6 mice per group; for (M)‐(P), *n* = 3 mice per group. *p < 0.05, **p < 0.01, and ***p < 0.001.

Furthermore, we analyzed the functional alterations of the gut microbiome by LEfSe for level‐three KEGG pathways between FMT_Pre_ and FMT_Post_ mice. Interestingly, we found that gut microbiota from the FMT_Post_ mice showed enrichment of butanoate metabolism and propanoate metabolism (Figure S9A, Supporting Information). We further measured the concentration of SCFAs in caecum samples. Compared with FMT_Pre_ mice, FMT_Post_ mice had significantly increased total SCFAs, including AA, PA, isobutyric acid (IBA), and isovaleric acid (IVA) (**Figure** **7**A,B; Figure S9B, Supporting Information). Furthermore, the mRNA expression levels of genes encoding tight‐junction proteins, including Zo1, Occludin, and Claudin1, and the immunohistochemical staining of these tight‐junction proteins in the colon revealed enhanced intestinal barrier integrity in FMT_Post_ mice compared to FMT_Pre_ mice (Figure 7C–I). With respect to inflammation, we observed significantly lower levels of pro‐inflammatory factors in FMT_Post_ mice than FMT_Pre_ mice, supported by the serum levels of LBP, IL‐1β, and IL‐18 (Figure 7J–L) and the ovarian mRNA levels of *Lbp, Il1b*, and *Il18* (Figure S9C, Supporting Information). Moreover, the TLR4/Myd88/NF‐κB/NLRP3/GSDMD signaling pathways in the ovarian tissues were also evaluated between FMT_Pre_ and FMT_Post_ mice by western blot. FMT_Post_ mice exhibited significantly reduced expression of TLR4, Myd88, and p‐p65/p65 compared to those in FMT_Pre_ mice (Figure 7M,O), accompanied by downregulation of downstream NLRP3 inflammasome (NLRP3/ASC/cleaved‐Caspase‐1) and gasdermin‐D activation (cleaved‐GSDMD) (Figure 7N,P), which potentially underlie the ovarian functional improvements by the post‐inulin microbiota. These results support the notion that gut microbiota from the post‐inulin intervention patients with PCOS have the capacity to mitigate PCOS‐like phenotypes in recipient mice.

## 3 Discussion

In this study, we demonstrated that inulin alleviated metabolic disorders and ovarian dysfunction associated with PCOS, which was mediated by an increase in the abundance of SCFAs‐producing bacteria in the gut. Notably, the most consistent alteration observed was the enrichment of beneficial bacteria with SCFAs‐producing capabilities, represented by *Bifidobacterium*, following inulin treatment in both patients with PCOS and a mouse model of PCOS. The finding was further validated in a FMT model, where gut microbiota from the inulin‐treated patients with PCOS improved metabolic and reproductive health in antibiotic‐treated recipient mice. The gut microbiota from the inulin‐treated patients with PCOS promoted SCFAs production, strengthened intestinal barrier integrity, and inhibited inflammatory responses in the recipient mice. These results highlight that the inulin‐modulated gut microbiome plays an important role in the prevention and treatment of PCOS.

Unhealthy dietary patterns, combined with hyperandrogenism, hyperinsulinemia, and chronic low‐grade inflammation, are widely recognized as metabolic risk factors related to PCOS.^[^ ^47^ ^]^ A meta‐analysis revealed that women with PCOS tend to consume less dietary fiber,^[^ ^23^ ^]^ while adequate dietary fiber intake has been shown to reduce body fat and improve glucose metabolism in patients with PCOS.^[^ ^48^ ^]^ Therefore, dietary intervention, an important aspect of a healthy lifestyle, is recommended as the cornerstone of treatment for all women with PCOS.^[^ ^9^ ^]^ Inulin, as a dietary fiber, has demonstrated benefits for various chronic metabolic diseases, with a recommended daily intake of 5–15 g for adults.^[^ ^22^ ^]^ Although several clinical studies have shown that inulin, or prebiotics and synbiotics containing inulin, can significantly reduce blood lipid levels and inflammatory markers in patients with PCOS,^[^ ^28^, ^29^, ^49^, ^50^ ^]^ the specific effects of inulin on the gut microbiome and its therapeutic mechanism for PCOS still remain unclear. In this study, we provide robust evidence supporting the causal relationship between clinical improvements in patients with PCOS and the restructured gut microbiota by inulin. Through transplantation of paired fecal microbiota from the patients with PCOS before and after inulin intervention into two groups of antibiotic‑treated mice, we demonstrated a direct causal link between gut microbiota and PCOS phenotypes.

Increasing evidence highlights the significance of community interactions within the gut ecosystem.^[^ ^51^, ^52^ ^]^ In this study, we incorporated ecological theories into the analysis of gut microbiota data in mice. From the microbial co‐abundance network analysis, we identified intricate relationships among microbial communities. Specifically, CAG12 and CAG16, represented by *Bifidobacterium* and Muribaculaceae, were enriched in NC and PCOS_Inu_ mice and exhibited synchronous fluctuations. In contrast, CAG2 and CAG32, represented by Lachnospiraceae and dominant in PCOS_Ctr_ mice, demonstrated a dynamically competitive relationship with CAG12. Lachnospiraceae is a diverse and controversial bacterial family,^[^ ^53^ ^]^ with mendelian randomization analysis revealing its detrimental effects on PCOS.^[^ ^54^, ^55^ ^]^ Moreover, HFD has been shown to induce a compensatory increase in Lachnospiraceae,^[^ ^56^ ^]^ consistent with our findings. In addition, Muribaculaceae, a dominant family in the mouse intestine, possesses an abundance of carbohydrate‐hydrolyzing enzymes, enabling it to utilize dietary fiber such as inulin as an energy source. Importantly, this family exhibits interspecific cross‐feeding relationships with *Bifidobacterium*,^[^ ^57^, ^58^ ^]^ which potentially serve as a key driver of their symbiotic relationship within CAG12 and CAG16. These findings underscore the need for greater attention to the complex interactions among gut microbiota in future studies.

Inulin intervention reduced the alpha diversity of the gut microbiota community in both PCOS mice and patients. It is noteworthy that microbiota richness and diversity should not be simplistically equated with gut health and stability.^[^ ^59^ ^]^ The gut environment, including transit time, stool consistency, and nutrient availability, is of significance in determining the gut microbiota richness.^[^ ^59^ ^]^ Polysaccharide‐based prebiotic interventions accelerate colonic transit, leading to decreased water reabsorption and looser stools, and finally reduce fecal microbial diversity,^[^ ^59^, ^60^ ^]^ which are consistent with our findings. However, from an ecological perspective, reduced microbial diversity may compromise the stability of the gut ecosystem in response to environmental perturbations, which merits long‐term evaluation in future investigations.

The dysbiosis of gut microbiota indued by DHEA and a high‐fat diet in a PCOS‐like mouse model was reversed by inulin consumption, leading to an increased abundance of SCFA‐producing community represented by *Bifdobacterium*. Similar outcomes were observed in the patients with PCOS.^[^ ^15^ ^]^ *Bifidobacterium*, a widely recognized probiotic prevalent throughout the human colon, is commonly used in foods and medicines.^[^ ^46^ ^]^ Our findings were supported by an animal study showing that inulin treatment increased *Bifidobacterium* in PCOS mice.^[^ ^61^ ^]^ In addition, supplementation with *Bifidobacterium lactis* has been shown to improve sex hormone levels in patients with PCOS by modulating gut microbiome,^[^ ^18^ ^]^ consistent with our observation of a negative correlation between *Bifidobacterium* abundance and testosterone levels in patients with PCOS.^[^ ^18^ ^]^ Furthermore, we observed a bifidogenic effect of inulin in FMT_Post_ mice which were colonized with gut microbiota from inulin‐treated PCOS human donors, as evidenced by the enrichment of *Bifidobacterium animalis* in these mice. Inulin also reduced the abundance of *Escherichia‐Shigella* in patients with PCOS. *Escherichia‐Shigella* is a conditional pathobiont that has been associated with metabolic diseases,^[^ ^62^, ^63^ ^]^ including PCOS.^[^ ^15^ ^]^ However, its relative abundance was low at baseline in our cohort, suggesting it may not be a key factor in the improvement of PCOS by inulin.

SCFAs play an important role in maintaining gut homeostasis.^[^ ^40^ ^]^ They can lower intestinal pH levels and stimulate the synthesis of antimicrobial peptides (e.g., defensins and lysozymes), which help prevent the overgrowth of pathobionts.^[^ ^64^ ^]^ Furthermore, SCFAs serve as an energy source for intestinal epithelia, activating the transcription and expression of genes encoding tight junction proteins that are essential for maintaining intestinal barrier integrity.^[^ ^65^, ^66^ ^]^ These mechanisms strongly support our findings, as both PCOS_Inu_ mice treated with inulin and FMT_Post_ mice receiving gut microbiota from the donors post‐inulin intervention exhibited significantly increased SCFA‐producing bacteria and SCFAs. These changes were accompanied by improved metabolic dysregulation, enhanced thermogenic activity, and better intestinal barrier integrity.

The impairment of the intestinal barrier is associated not only with intestinal disorders but also with systemic diseases. The dysbiosis of gut microbiota (DOGMA) hypothesis of PCOS proposes that intestinal dysbiosis and hyperpermeability result in the leakage of pro‐inflammatory substances, such as LPS and bacterial components, from the gut lumen into the circulation.^[^ ^41^ ^]^ This triggers immune activation and inflammatory responses, which negatively affect metabolism.^[^ ^67^ ^]^ Studies have shown impaired intestinal barrier in PCOS mice induced by DHEA and a high‐fat diet^[^ ^68^ ^]^ and in PCOS rats induced by letrozole.^[^ ^69^ ^]^ Our study is consistent with these findings but provides deeper insights into the underlying molecular mechanisms, including assessment of LPS‐binding protein and proinflammatory cytokines (IL‐1β and IL‐18) in the circulation. Increasing evidence suggests that chronic low‐grade inflammation is a hallmark of PCOS.^[^ ^42^, ^70^, ^71^, ^72^ ^]^ Notably, elevated levels of proinflammatory cytokines, including IL‐1β and IL‐18, have been detected in the follicular fluid of patients with PCOS.^[^ ^73^, ^74^, ^75^ ^]^ Moreover, a recent study reported a strong association between the NLRP3 inflammasome‐pyroptosis pathway and ovarian dysfunction in PCOS,^[^ ^76^ ^]^ with overexpression of pyroptosis‐related proteins observed in the ovaries of PCOS animal models.^[^ ^73^, ^77^, ^78^, ^79^ ^]^ Here, we showed that inulin or inulin‐modified gut microbiota effectively reduced intestinal permeability, preventing LPS entry into the bloodstream and downregulating the inflammation response in the ovary. These findings provide novel insights into how inulin alleviates ovarian inflammation in PCOS. However, further precision studies are needed to fully elucidate these mechanisms.

In conclusion, we investigated how inulin modulates the composition of gut microbiota and improves the clinical outcome in patients with PCOS. To our knowledge, this has not been reported before. Our results indicate that inulin selectively promotes the growth of beneficial bacteria such as *Bifidobacterium*, thereby alleviating the dysbiosis observed in patients with PCOS. Furthermore, inulin enhances the abundance of SCFAs‐producing bacteria, which ameliorate PCOS‐associated symptoms, including ovulatory dysfunction, hyperandrogenism, glucolipid metabolism disorders, and chronic low‐grade inflammation, through interactions with various host cells. Our study further demonstrates the critical role of gut microbiota in the progression of PCOS, and highlights inulin as a promising therapeutic strategy for rebalancing gut microbiota homeostasis and managing PCOS clinically.

## 4 Limitations of the Study

Our findings in both humans and mice support the notion that inulin alleviates PCOS by modulating gut microbiota. However, there are some limitations in the study. First, our human study employed a prospective, self‐controlled before‐and‐after trial design. Although this approach eliminated confounding factors related to intraindividual differences in gut microbiota, the strength of the clinical evidence is limited. Ideally, a randomized, placebo‐controlled, double‐blind trial should be conducted to strengthen the conclusions. Second, in the FMT experiment, although the gut microbiota from pre‐ and post‐inulin intervention inherently provided a contrast, demonstrating the beneficial effects of inulin, the microbiota‐depleted recipient mice were only given the high‐fat diet without DHEA injection. This decision was based on our preliminary experiments showing that antibiotic‐treated mice receiving continuous DHEA injection exhibited poor physical conditions, making them unsuitable for further experimentation. Nevertheless, a high‐fat diet alone can induce PCOS‐related metabolic disorders.^[^ ^80^ ^]^ Lastly, we currently were unable to isolate the core strain group represented by *Bifidobacterium* from the feces of patients with PCOS after inulin intervention, which would have allowed us to precisely verify their roles and mechanisms in improving PCOS. Addressing these limitations will be the direction of our future studies.

## Conflict of Interest

The authors declare no competing interests.

## Author Contributions

L.G. and X.Y. contributed equally to this work. R.W. and M.C. designed and supervised the study. L.G., J.N., and D.Z. contributed to the enrollment and follow‐up of the study subjects and the collection of fecal samples. L.G., J.N., D.Z., and M.Y. performed the animal experiments. L.G., X.Y., and X.R. analyzed and interpreted data. L.G. and X.Y. drafted the manuscript. L.W., R.W., and M.C. edited and revised the manuscript. All authors approved the final version of the manuscript.

## Supporting information



Supporting Information

Supplementary Table

## Data Availability

The data that support the findings of this study are available from the corresponding author upon reasonable request.
