# Supplementary material for: Gut Microbiota Modulation by Inulin Improves Metabolism and Ovarian Function in Polycystic Ovary Syndrome
Source: Adv Sci (Weinh). 2025 Apr 7;12(20):2412558. doi: 10.1002/advs.202412558 (PMC12120758; doi:10.1002/advs.202412558)
Supplement: Supplementary file 1 — Supporting Information [file ADVS-12-2412558-s001.docx]

**Supplementary information**

**Supplementary Figure S1. Metabolic and ovarian dysfunction in a PCOS-like mouse model induced by DHEA and high-fat diet. Related to Figure 1.**


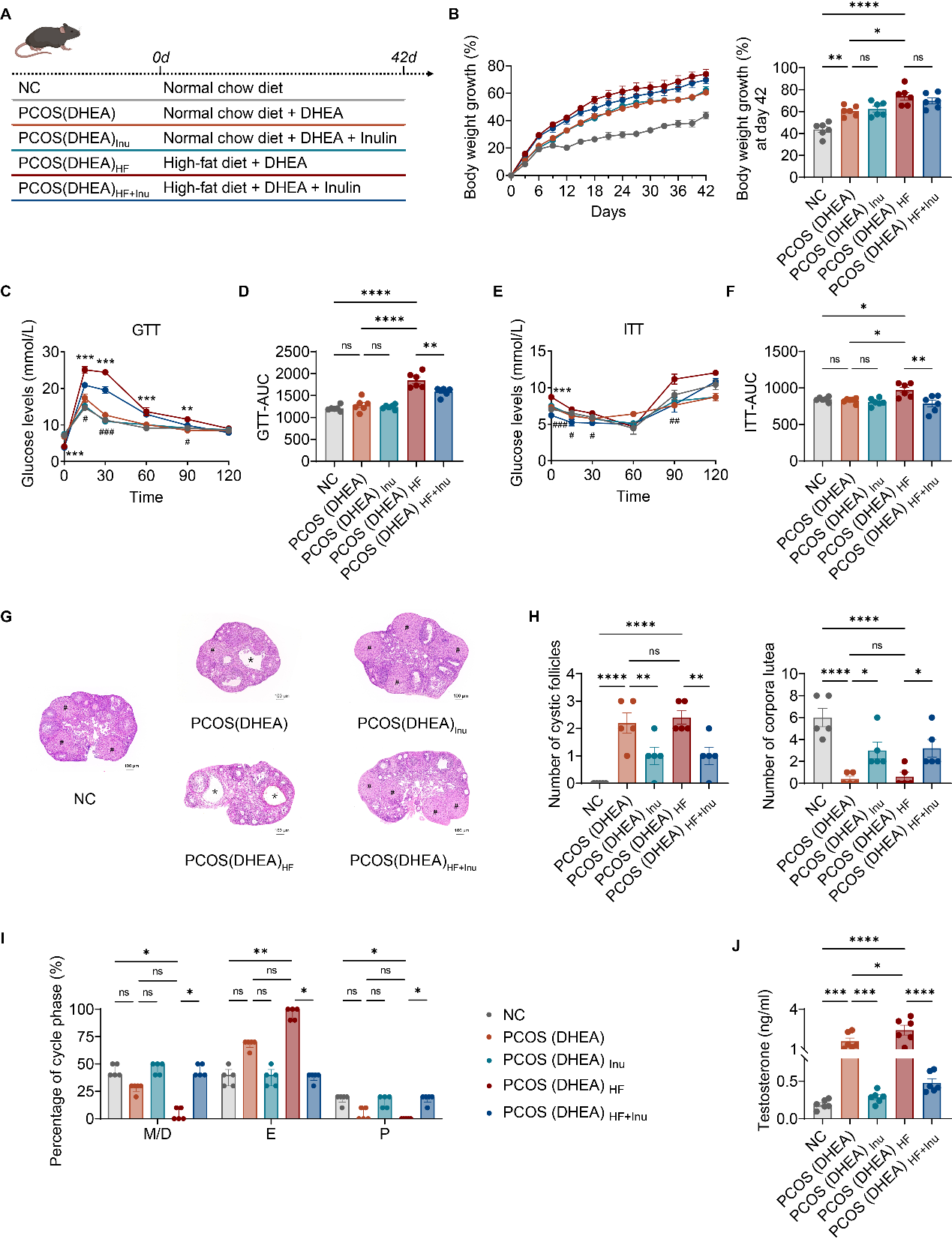


(A) Schematic diagram of the mouse model design. NC, normal control mice fed a chow diet and normal drinking water; PCOS(DHEA), mice injected with DHEA and fed a chow diet and normal drinking water; PCOS(DHEA)_Inu_, mice injected with DHEA, fed a chow diet and treated with 4% (w/v) inulin in drinking water; PCOS(DHEA)_HF_, mice injected with DHEA and fed a HFD and normal drinking water; PCOS(DHEA)_HF+Inu_, mice injected with DHEA, fed a HFD, and treated with 4% (w/v) inulin in drinking water. (B) Percentage growth in body weight during the experimentation. (C and D) Blood glucose levels in GTT (C) and AUC of GTT (D). (E and F) Blood glucose levels in ITT (E) and AUC of ITT (F). (G) Representative H&E-stained histological sections of ovaries (5×, scale bar = 100 μm). ＊indicates cystic follicle; # indicates corpora luteum. (H) Number of cystic follicles and corpora lutea. (I) Quantitative analysis of each phase in estrous cycles. P, proestrus; E, estrus; M, metestrus; D, diestrus. (J) Serum testosterone levels. The data are shown as the mean ± SEM and statistical significance was analyzed by one-way ANOVA with Tukey’s multiple comparisons test. For (A) to (F), and (J), n = 6 mice per group; for (G) to (I), n = 5 mice per group. For (C) and (E), * indicates NC vs. PCOS(DHEA)_HF_; # indicates PCOS(DHEA)_HF_ vs. PCOS(DHEA)_HF+Inu_. * P < 0.05, ** P < 0.01, *** P < 0.001 and **** P < 0.0001; # P < 0.05, ## P < 0.01, and ### P < 0.001; ns means no statistical difference.

**Supplementary Figure S2. Effects of different inulin doses on glucose metabolism in PCOS mice. Related to Figure 1.**


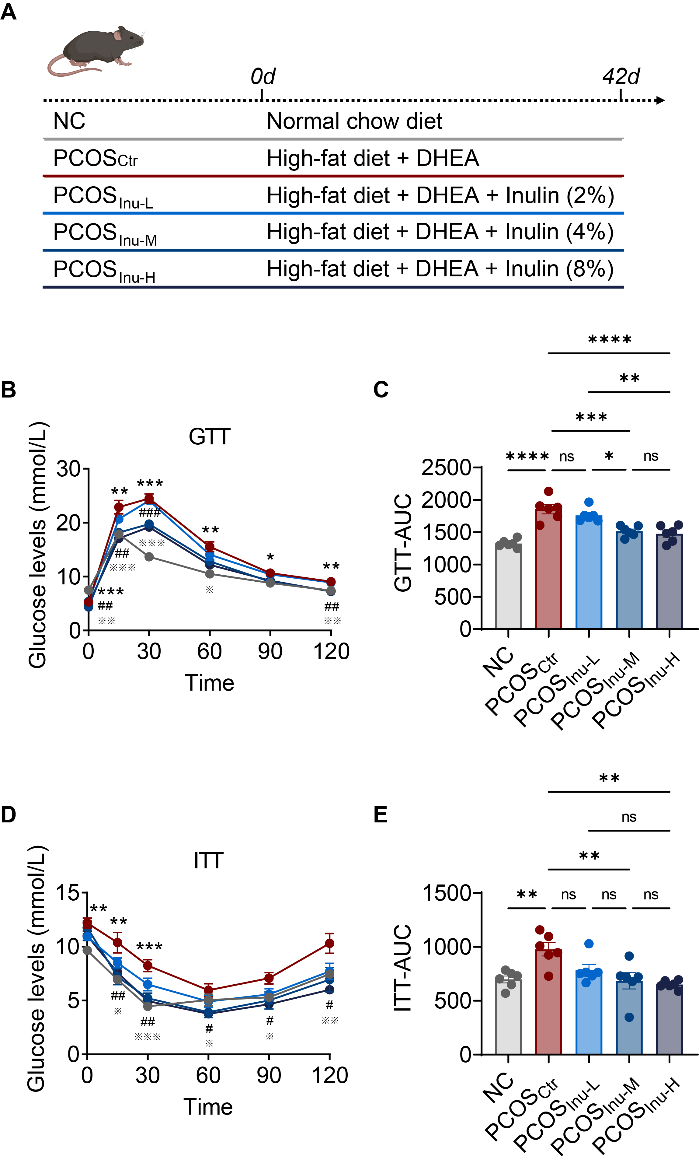


(A) Schematic diagram of mice trial. NC, normal control mice provided a chow diet and normal drinking water; PCOS_Ctr_, PCOS-like mice induced by DHEA and HFD with normal drinking water; PCOS_Inu-L_, PCOS_Inu-M_, and PCOS_Inu-H_, mice induced by DHEA and HFD, treated with low (2% w/v), medium (4% w/v) and high (8% w/v) inulin in drinking water, respectively. (B and C) Blood glucose levels in GTT (B) and AUC of GTT (C). (D and E) Blood glucose levels in ITT (D) and AUC of ITT (E). The data are shown as the mean ± SEM and statistical significance was analyzed by one-way ANOVA with Tukey’s multiple comparisons test (n=6 mice per group). * indicates NC vs. PCOS_Ctr_; # indicates PCOS_Ctr_ vs. PCOS_Inu-M_; ※ indicates PCOS_Ctr_ vs. PCOS_Inu-H_. * P < 0.05, ** P < 0.01, *** P < 0.001 and **** P < 0.0001; # P < 0.05, ## P < 0.01, and ### P < 0.001; ※ P < 0.05, ※※ P < 0.01, and ※※※ P < 0.001; ns means no statistical difference.

**Supplementary Figure S3. The differential analysis of gut microbial communities and SCFAs in PCOS mice. Related to Figure 2.**


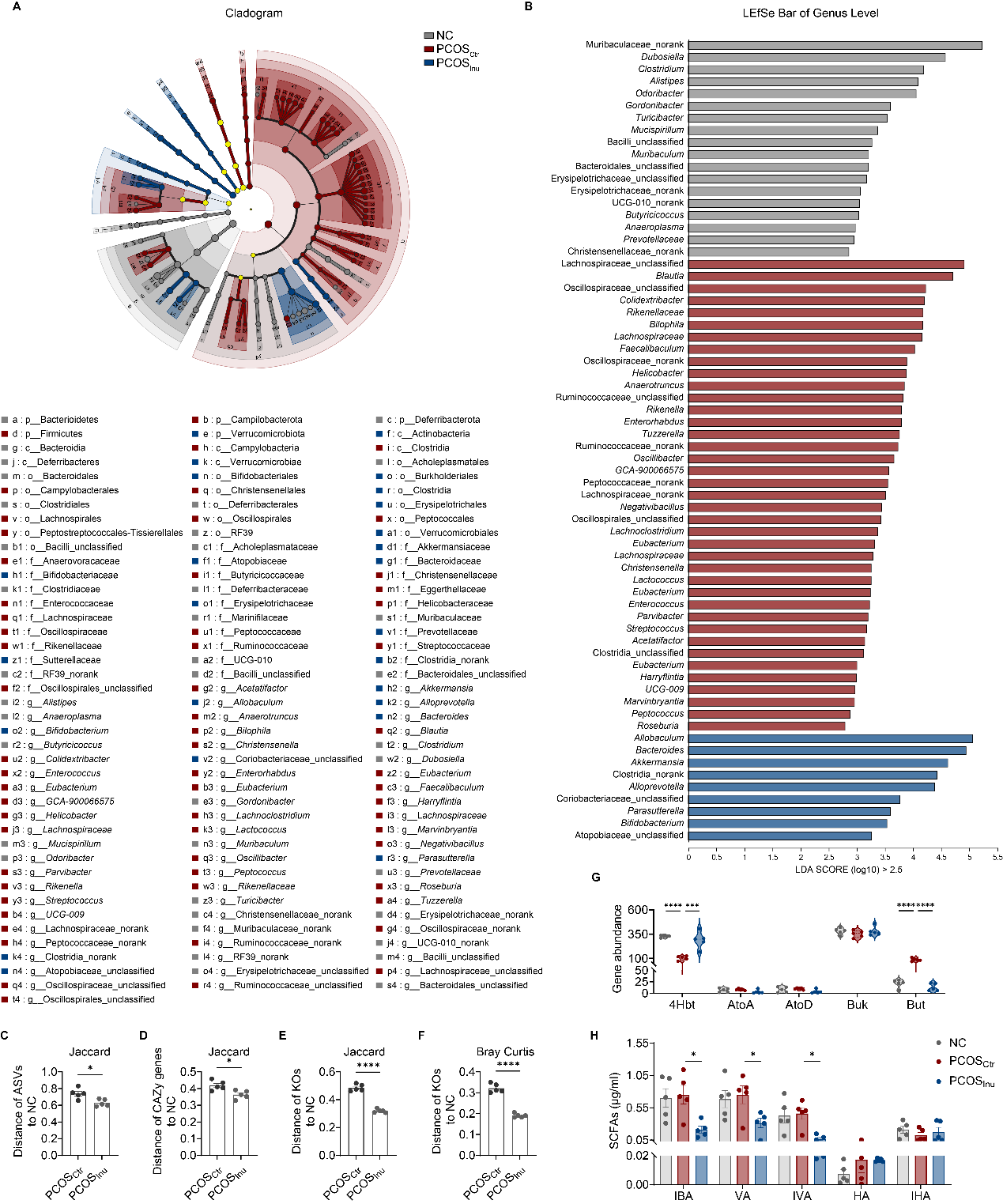


(A) The taxonomic cladogram analyzed by Linear discriminant analysis effect size (LEfSe) showing differences of gut microbiota among NC, PCOS_Ctr_, and PCOS_Inu_ mice. (B) The bar chart of genus level in NC, PCOS_Ctr_, and PCOS_Inu_ mice with LDA score > 2.5 based on LEfSe. The colour of taxa indicates the predominance of this taxa in the corresponding colour group (NC in grey, PCOS_Ctr_ in red, PCOS_Inu_ in blue; n = 5 mice per group). (C) Comparison of distances of ASVs between samples of NC mice and samples of PCOS_Ctr_ or PCOS_Inu_ mice, based on the Jaccard distance. (D) Comparison of distances of CAZy family genes between samples of NC mice and samples of PCOS_Ctr_ or PCOS_Inu_ mice, based on the Jaccard distance. (E and F) Comparison of distances of KOs between samples of NC mice and samples of PCOS_Ctr_ or PCOS_Inu_ mice, based on the Jaccard (E) and Bray-Curtis (F) distance. (G) The abundance of butyrate production corresponding genes (displayed by protein names): 4Hbt, butyryl–coenzyme A (butyryl-CoA): 4-hydroxybutyrate CoA transferase; Ato, butyryl-CoA: acetoacetate CoA transferase (AtoA: alpha subunit, AtoD: beta subunit); Buk, butyrate kinase; But, butyryl-CoA: acetate CoA transferase. (H) The concentration of the other five additional SCFAs in the caecum of NC, PCOS_Ctr_, and PCOS_Inu_ mice, including isobutyric acid (IBA), valeric acid (VA), isovaleric acid (IVA), hexanoic acid (HA), and isohexanoic acid (IHA). The data in (C) to (F) and (H) are shown as the mean ± SEM; data in (G) are shown as violin plots with the median, interquartile ranges (IQRs), and min/max values. Two-tailed Student’s t test in (C) to (F); one-way ANOVA with Tukey’s multiple comparisons test in (G) and (H) (n = 5 mice per group). *P < 0.05, *** P < 0.001, and **** P < 0.0001.

**Supplementary Figure S4.** **Correlation analysis between the abundance of inulin-enriched genera and genes involved in SCFAs production. Related to Figure 2.**


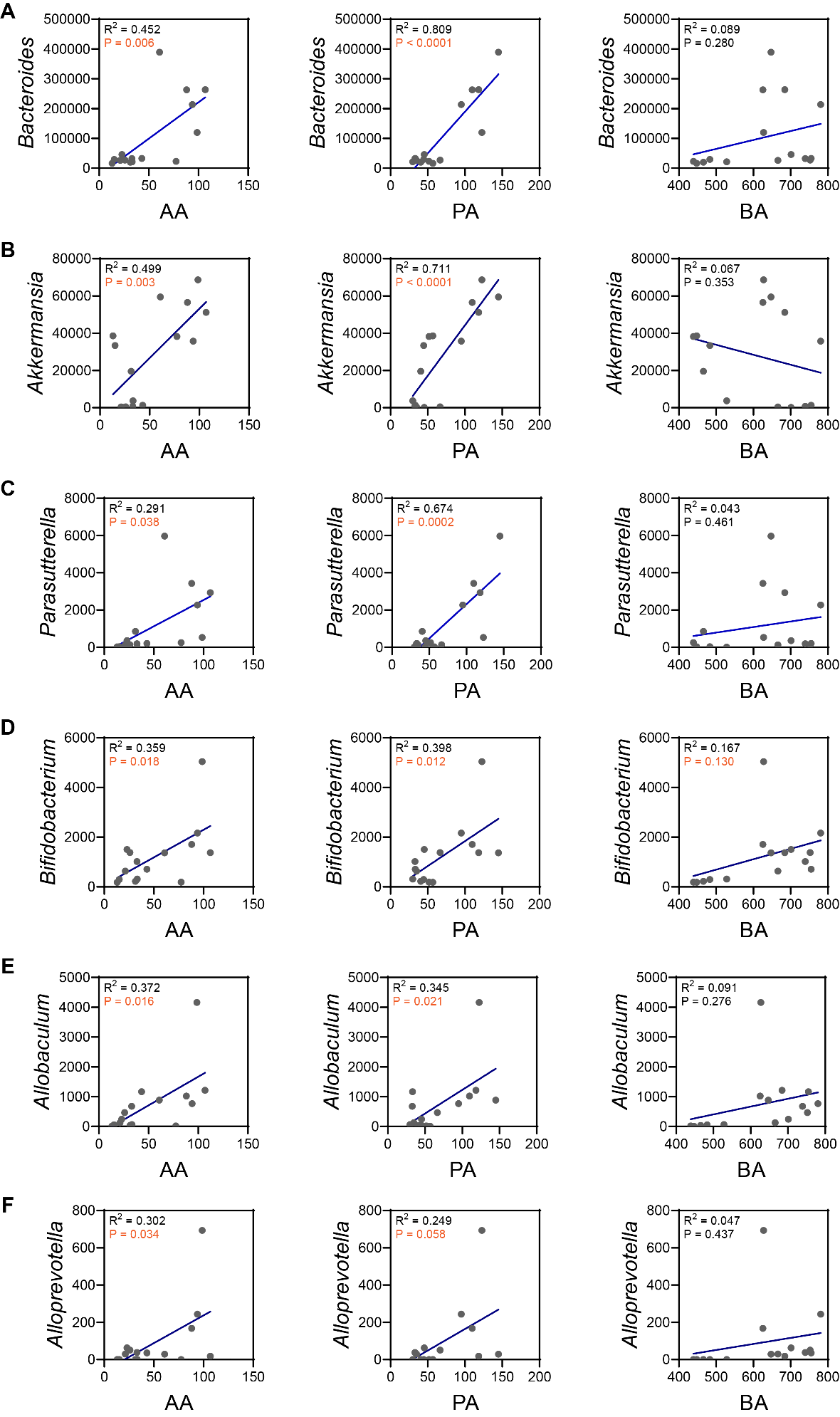


Inulin-enriched genera including *Bacteroides* (A), *Akkermansia* (B), *Parasutterella* (C), *Bifidobacterium* (D), *Allobaculum* (E), and *Alloprevotella* (F). The data are presented as a scatterplot with a fitted regression line, accompanied by the Pearson correlation coefficient and P-value.

**Supplementary Figure S5. Volcano plots of differentially expressed genes from** **comprehensive antibiotic resistance database (CARD) and virulence factors database (VFDB) in PCOS mice. Related to Figure 2.**


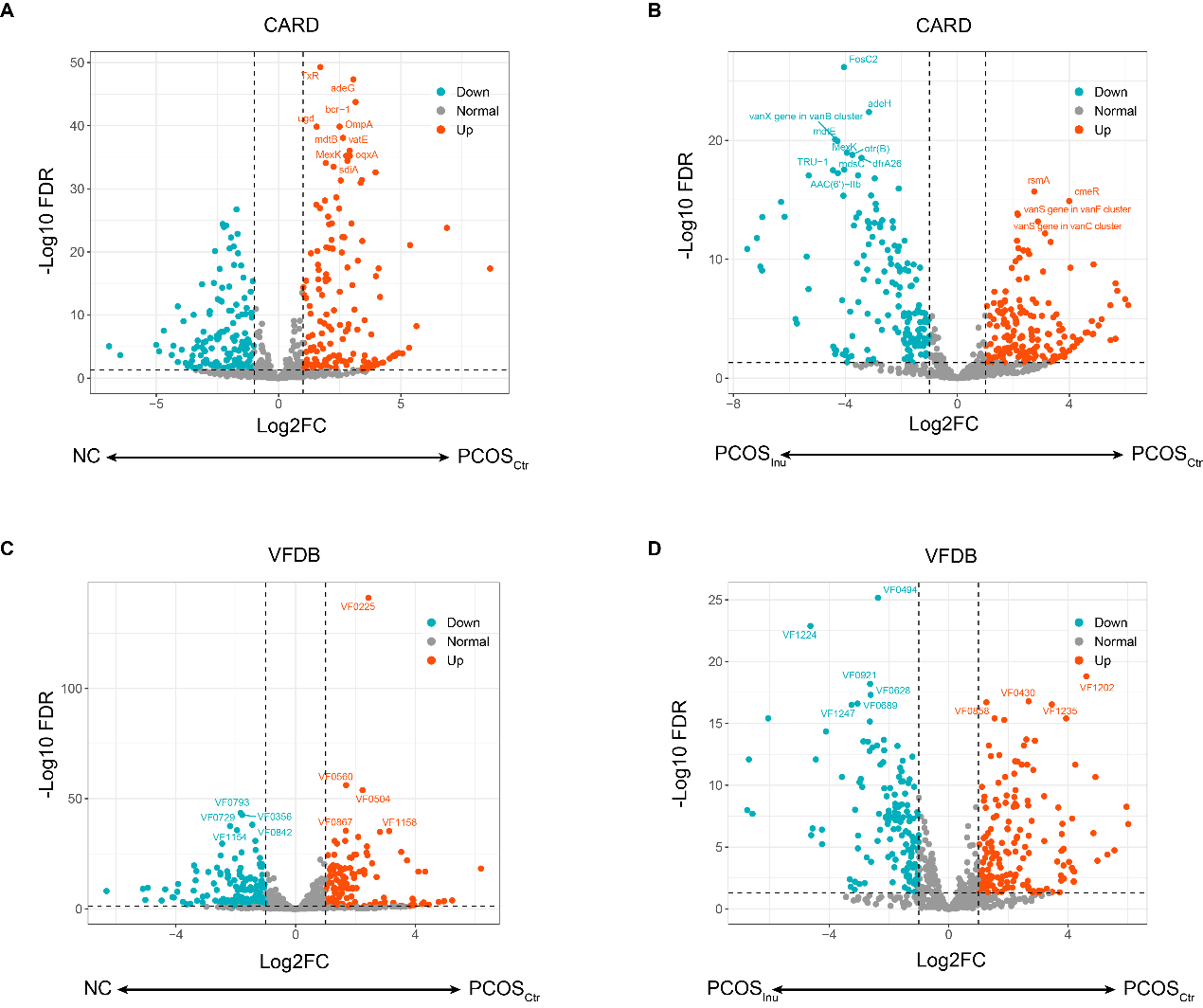


(A and B) Volcano plots of differentially expressed antibiotic resistance genes (ARGs) from CARD between the PCOS_Ctr_ and NC mice (A) as well as between the PCOS_Ctr_ and PCOS_Inu_ mice (B). (C and D) Volcano plots of differentially expressed virulence factor genes (VFGs) from VFDB between the PCOS_Ctr_ and NC mice (C) as well as between the PCOS_Ctr_ and PCOS_Inu_ mice (D). N = 5 mice per group.

**Supplementary Figure S6. The LEfSe analysis for gut microbial functional pathways in PCOS mice. Related to Figure 2 and 4.**


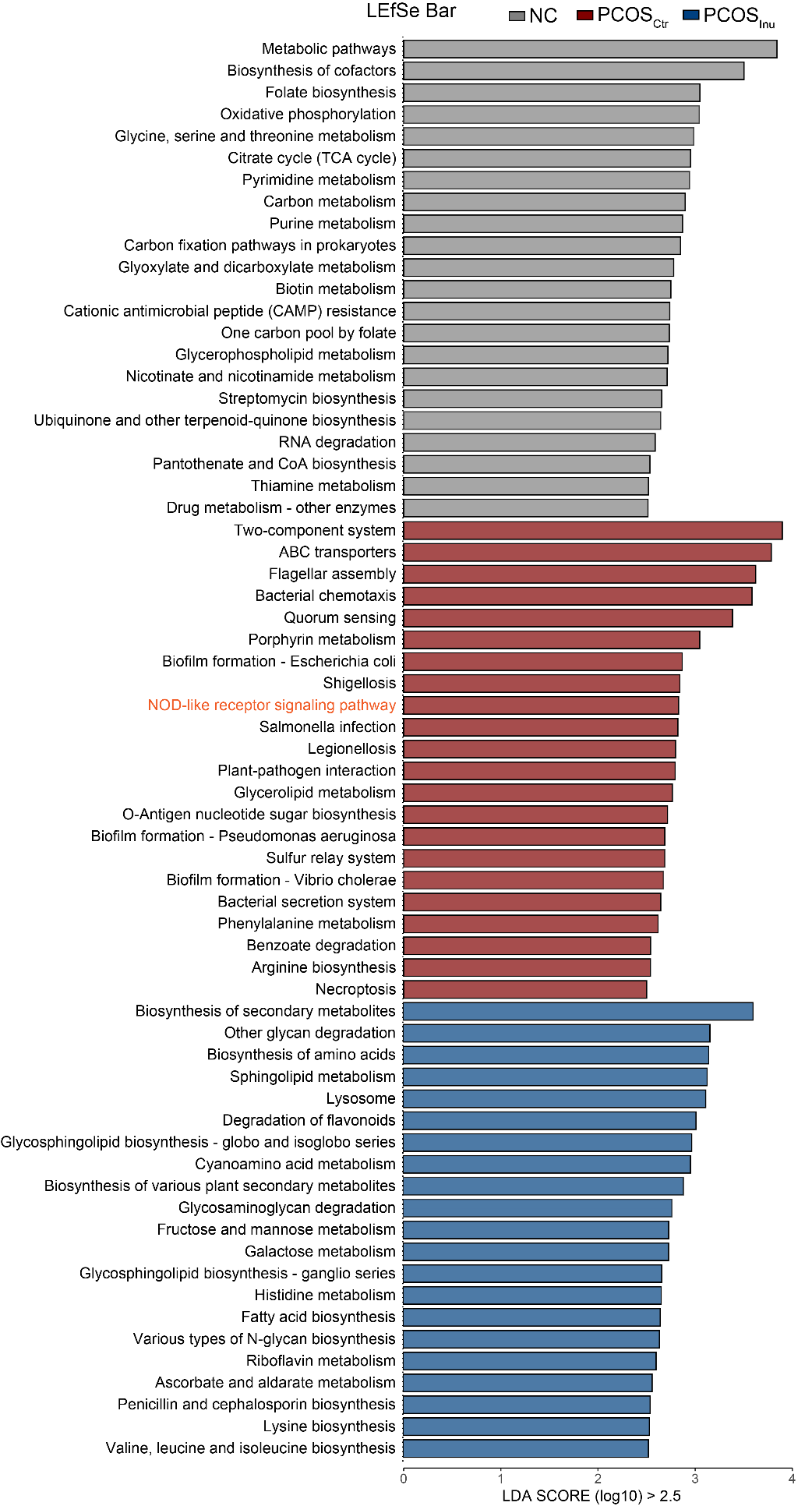


**Supplementary Figure S7.** **Lipopolysaccharide (LPS) reverses the protective effects of inulin in PCOS mice. Related to Figure 4.**


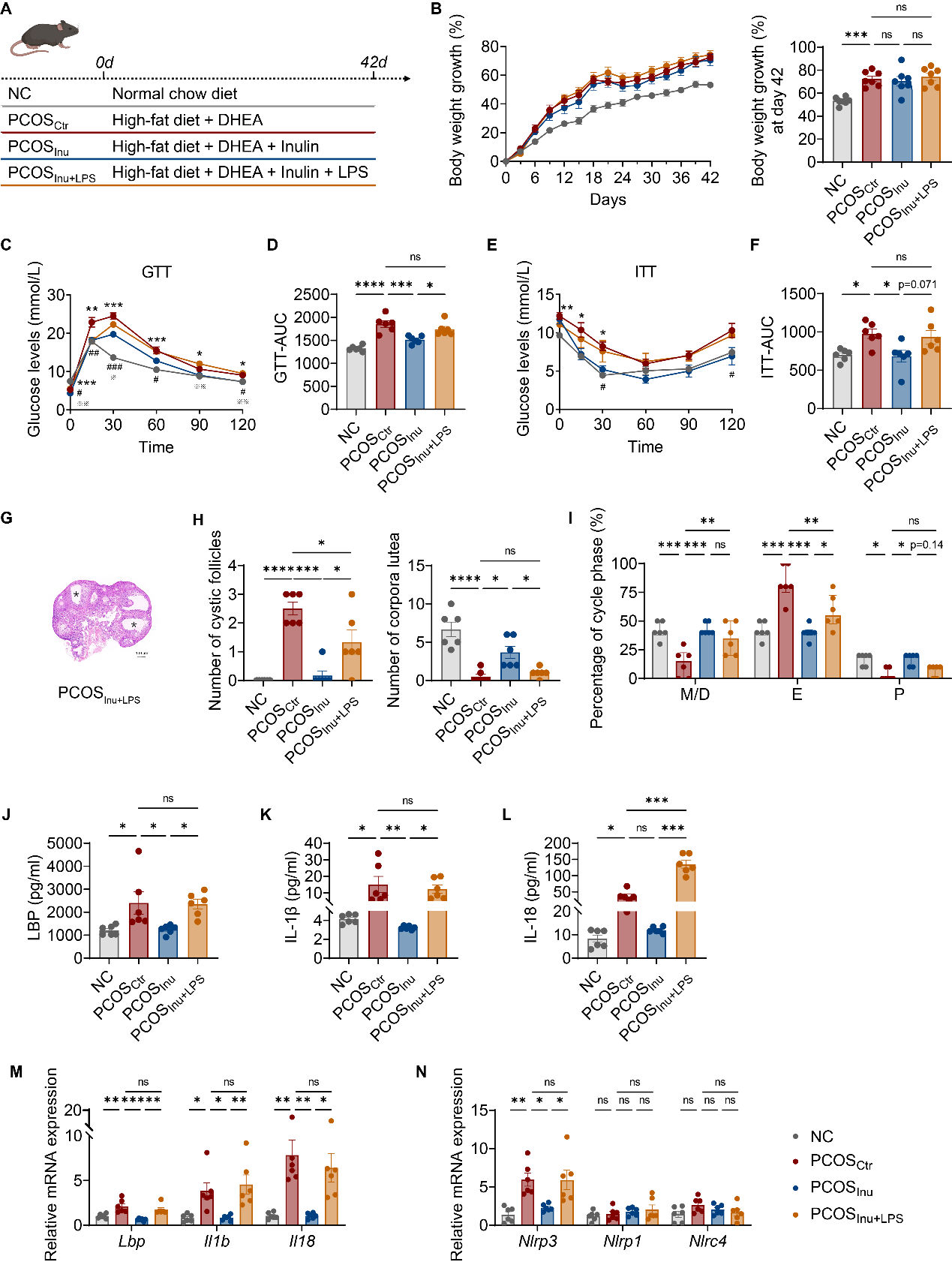


(A) Schematic diagram of mice trial. NC, normal control mice with a chow diet and normal drinking water; PCOS_Ctr_, PCOS-like mice induced by DHEA and HFD with normal drinking water; PCOS_Inu,_ induced by DHEA and HFD, treated with inulin in drinking water; PCOS_Inu+LPS,_ induced by DHEA and HFD, treated with inulin in drinking water and intraperitoneal injection of LPS. (B) Percentage growth in body weight during the experimentation. (C and D) Blood glucose levels in GTT (C) and AUC of GTT (D). (E and F) Blood glucose levels in ITT (E) and AUC of ITT (F). (G) Representative H&E-stained histological sections of ovaries (5×, scale bar = 100 μm) in PCOS_Inu+LPS_ mice. ＊indicates cystic follicle. (H) Number of cystic follicles and corpora lutea. (I) Quantitative analysis of each phase in estrous cycles. P, proestrus; E, estrus; M, metestrus; D, diestrus. (J-L) Serum levels of LBP (J), IL-1β (K), and IL-18 (L). (M) RT-qPCR analysis of mRNA expression levels of *Lbp, Il1b,* and *Il18* in the ovary. (N) RT-qPCR analysis of mRNA expression levels of *Nlrp3, Nlrp1,* and *Nlrc4* in the ovary. The data are shown as the mean ± SEM and statistical significance was analyzed by one-way ANOVA with Tukey’s multiple comparisons test (n = 6 per group). For (C) and (E), * indicates NC vs. PCOS_Ctr_; # indicates PCOS_Ctr_ vs. PCOS_Inu_; ※ indicates PCOS_Inu+LPS_ vs. PCOS_Inu_. * P < 0.05, ** P < 0.01, *** P < 0.001 and **** P < 0.0001; # P < 0.05, ## P < 0.01, and ### P < 0.001; ※ P < 0.05 and ※※ P < 0.01; ns means no statistical difference.

**Supplementary Figure S8. The bifidogenesis effect of inulin in patients with PCOS. Related to Figure 5.**


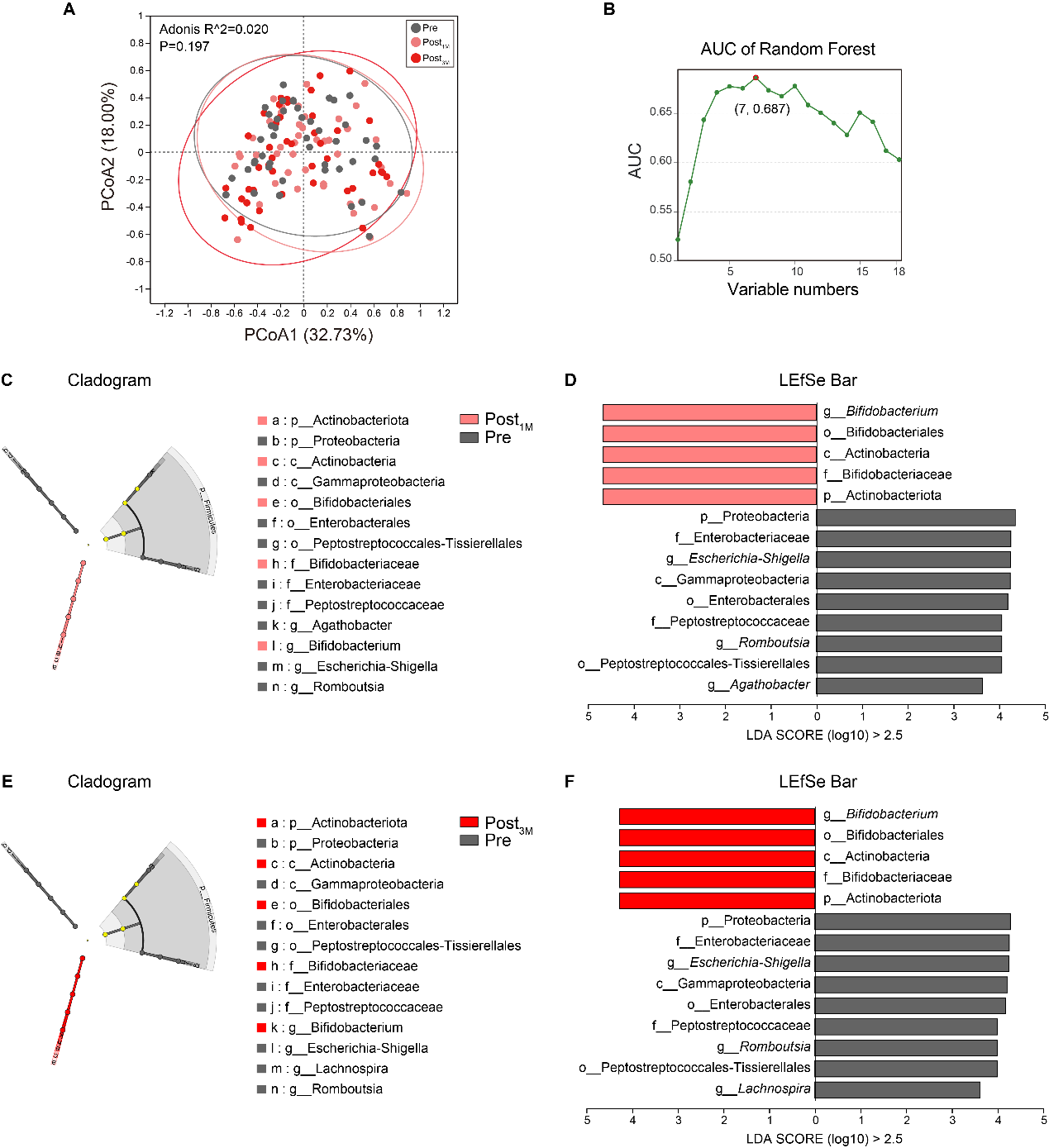


(A) Overall structure of gut microbiota in Pre, Post_1M_, and Post_3M_ groups. PCoA based on the weighted UniFrac distance of ASVs and between-group differences determined by Adonis analysis. (B) Area under the curve (AUC) of receiver operating characteristic curve in the random forest model. (C-F) The taxonomic cladogram and bar chart of LDA score > 2.5 based on LEfSe analysis showing differences of gut microbiota between the Pre and Post_1M_ groups (C and D) as well as the Pre and Post_3M_ groups (E and F). The colour of taxa indicates the predominance of this taxa in the corresponding colour group (Pre in grey, Post_1M_ in pink, Post_3M_ in red; n = 45 patients per group).

**Supplementary Figure S9. Differences in gut microbial function, SCFAs and ovarian inflammation in mice receiving FMT. Related to Figure 7.**


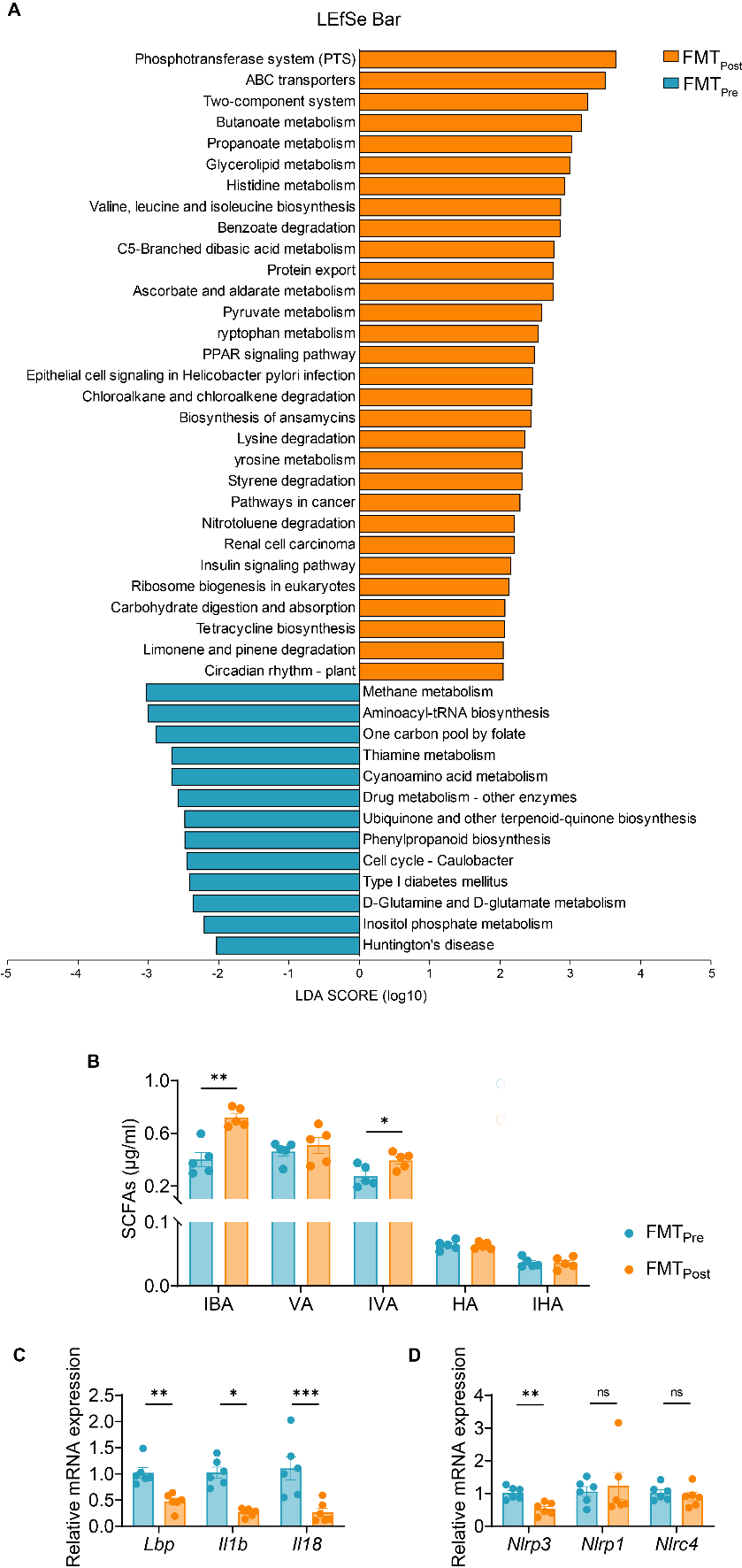


(A) The LEfSe analysis for differential pathways with the involvement of gut microbiota between FMT_Pre_ and FMT_Post_ mice. (B) The concentration of the other five additional SCFAs in the caecum of FMT_Pre_ and FMT_Post_ mice, including IBA, VA, IVA, HA, and IHA. (C) RT-qPCR analysis of mRNA expression levels of *Lbp, Il1b,* and *Il18* in the ovary. (D) RT-qPCR analysis of mRNA expression levels of *Nlrp3, Nlrp1,* and *Nlrc4* in the ovary. The data are shown as the mean ± SEM and statistical significance was analyzed by two-tailed Student’s t test. For (A) and (B), n = 5 mice per group; for (C) and (D), n = 6 mice per group. *P < 0.05, **P < 0.01, and ***P < 0.001; ns means no statistical difference.

**Materials and Methods**

**Human participants**

The human study was approved by the Ethics Committee of Shanghai First Maternity and Infant Hospital (Ethics number: KS2304) and registered in the Chinese Clinical Trail Registry (Registration number: ChiCTR2300072401). The participants were recruited from the Centre for Assisted Reproduction at Shanghai First Maternity and Infant Hospital from June 2023 to November 2023. All eligible participants signed the written informed consent.

PCOS diagnose was according to the 2003 Rotterdam criteria^[1]^, at least two of the following criteria were met: 1. oligo-ovulation and/or anovulation; 2. polycystic ovary morphology under ultrasound; 3. clinical and/or biochemical signs of hyperandrogenism. The diagnosis of PCOS excludes other etiologies for hyperandrogenemia and ovulatory disorder, including Cushing's syndrome, 21-hydroxylase deficiency, ovarian and adrenal tumors, congenital adrenal hyperplasia, thyroid dysfunction, and hyperprolactinemia. The enrolled subjects were between 18 and 35 years of age with BMI between 18.5 and 30 kg/m^2^. The additional exclusion criteria were: use of probiotics, prebiotics, antibiotics, and other medical treatments for metabolism and ovulation within the past 3 months; combination of diarrhea, constipation, gastritis, and other gastrointestinal conditions, or diabetes mellitus, abnormal liver and kidney function, and other metabolic diseases; in pregnancy or lactation.

**Implementation process**

The use of inulin dose and duration were based on the published studies^[2, 3, 4]^, the enrolled subjects in this study daily received 10 g of inulin (2022101202001NH, Weinihao, China), dissolved in drinking water, for three months. This dose was sufficient for health effects and without noticeable adverse gastrointestinal side effects. The study subjects were required to maintain their lifestyle and dietary habits and not to use prebiotics, probiotics, antibiotics, and other medications during the intervention period. The researchers followed up the subjects' conditions regularly, to ensure the consumption of inulin and assessed the occurrence of abdominal distension, diarrhea and other adverse side effects, the use of other medications, as well as the presence of pregnancy.

Clinical parameters were assessed at baseline and at the end of study, including basic sex hormone levels (testosterone, DHEAs, AMH); glucose metabolism indices (fasting blood glucose (FBG) and insulin (FIN), oral glucose (75g) tolerance test, HbA1c, HOMA-IR calculated by FBG ×FIN/22.5); as well as serum lipid panels (total cholesterol, triglyceride, high-density lipoprotein, low-density lipoprotein). Body weight was measured without shoes and coats and the BMI was calculated by body weight divided by height squared (kg/m^2^).

To investigate the dynamic changes of gut microbiota, we collected the fecal samples from the study subjects at different time points – before inulin intervention, 1 month and 3 months after inulin intervention. The study subjects were well instructed with the usage of the fecal sampler and the fresh sample was placed in a sterile tube containing preservation solution and stored at -80 °C, at earliest possible time, until assayed.

**Animal models**

**Mice**

Three-week-old female C57BL/6J mice were purchased from Charles River Laboratories (Beijing, China) and kept in a specific-pathogen-free barrier animal facility of Tongji University, Shanghai, China, with free access to water and food, under a 12-hour light-dark cycle (lights on at 07:00 am) and controlled conditions of temperature (22.0 ± 2°C) and humidity (40-70%). The mice underwent one week acclimation prior to the experiment. All procedures using the animals were approved by the Ethics Committee of Laboratory Animal Research Center, Tongji University (Ethics number: TJBG02323102 and TJBG02324103).

**PCOS-like mouse model trials**

**Trial-1**

To induce PCOS-like mouse model, mice were randomly assigned into 5 groups: (1) NC group, normal control mice daily injected subcutaneously (s.c.) with 100 μl sesame oil (S28312, Abcone, China) and fed a chow diet (1010058, Xietong Shengwu, China) and normal drinking water; (2) PCOS(DHEA) group, mice injected s.c. daily with dehydroepiandrosterone (DHEA, 6 mg per 100 g body weight^[5]^, dissolved in 100 μl of sesame oil; HY-14650, MedChemExpress, USA) and fed a chow diet and normal drinking water for 21 days; (3) PCOS(DHEA)_Inu_ group, mice injected s.c. daily with DHEA, fed a chow diet and treated with 4% (w/v) inulin (2022101202001NH, Weinihao, China) in drinking water for 21 days; (4) PCOS(DHEA)_HF_ group, mice injected s.c. daily with DHEA, fed a high-fat diet (HFD, 60% kcal fat^[6]^; D12492, Research Diets, USA) and normal drinking water for 21 days; (5) PCOS(DHEA)_HF+Inu_ group, mice injected s.c. daily with DHEA, fed a HFD and treated with 4% (w/v) inulin in drinking water for 21 days. The daily dosage of inulin supplementation was estimated to be approximately 0.2 g^[7, 8]^, which was calculated based on the daily water intake of nearly 5 ml per mouse.

**Trial-2**

To investigate the effects of different dosages of inulin on PCOS-like mice, mice were randomly assigned into 5 groups: (1) NC group, normal control mice fed a chow diet and normal drinking water; (2) PCOS_Ctr_ group, mice injected s.c. daily with DHEA, fed a HFD and normal drinking water for 21 days; (3) PCOS_Inu-L_ group, mice injected s.c. daily with DHEA, fed a HFD and treated with 2% (w/v) inulin in drinking water for 21 days; (4) PCOS_Inu-M_ group, mice injected s.c. daily with DHEA, fed a HFD and treated with 4% (w/v) inulin in drinking water for 21 days; (5) PCOS_Inu-H_ group, mice injected s.c. daily with DHEA, fed a HFD and treated with 8% (w/v) inulin in drinking water for 21 days.

**Trial-3**

After the determination of PCOS-like mouse model induced by DHEA and HFD and the appropriate dosage of inulin, to investigate the comprehensive therapeutic effects of inulin on PCOS, the mice were randomly assigned to 3 groups: (1) NC group, normal control mice fed a chow diet and normal drinking water; (2) PCOS_Ctr_ group, mice injected s.c. daily with DHEA, fed a HFD and normal drinking water for 21 days; (3) PCOS_Inu_ group, mice injected s.c. daily with DHEA, fed a high-fat diet and treated with 4% (w/v) inulin in drinking water for 21 days.

**Trial-4**

To investigate the relationship between inulin and inflammation in PCOS-like mice, mice were randomly assigned to 4 groups: (1) NC group, normal control mice fed a chow diet and normal drinking water; (2) PCOS_Ctr_ group, mice injected s.c. daily with DHEA, fed a HFD and normal drinking water for 21 days; (3) PCOS_Inu_ group, mice injected s.c. daily with DHEA, fed a HFD and treated with 4% (w/v) inulin in drinking water for 21 days; (4) PCOS_Inu+LPS_ group, mice injected s.c. daily with DHEA, intraperitoneally administered lipopolysaccharide (LPS, 100 µg/kg body weight^[9]^, L2630 from *Escherichia coli* 0111:B4, Sigma-Aldrich, USA) every three days, fed a HFD and 4% (w/v) inulin in drinking water for 21 days.

In all the PCOS-like mouse model trials, following the initial 21-day period, the daily DHEA injection was shifted to once every three days to maintain the persistent effect of exogenous androgens, with an additional 21-day observation period. The body weight of mice was recorded every three days. Body weight growth (%) = (end weight – original weight)/original weight × 100%.

**Fecal microbiota transplantation (FMT)**

Freshly pooled fecal samples from three randomly selected patients with PCOS before and after inulin intervention were prepared as previously described^[10]^. After one-week of acclimation, the mice were administered a cocktail of antibiotics (1 g/L of ampicillin, 1 g/L of metronidazole, 1 g/L of neomycin, and 0.5 g/L of vancomycin) (Aladdin, Shanghai, China) in drinking water for two weeks^[11]^. The administration of antibiotics was discontinued three days before the FMT experiment. Subsequently, the mice were randomly divided into 2 groups: FMT_Pre_ and FMT_Post_ group, referring the mice gavaged with the pooled fecal suspension (100 μL) from the donors before and after inulin intervention, respectively. FMT was given twice a week for three weeks. All the mice were fed with a HFD after the initiation of FMT. The body weight of mice was recorded every three days. Body weight growth (%) = (end weight – original weight)/original weight × 100%.

**16S rRNA gene sequencing and data analysis**

Fecal samples of patients and mice in Trial-3 were both extracted genomic DNA using the Stool DNA Extraction kit (Majorbio, Shanghai, China). The V3–V4 region of the bacterial 16S rRNA gene was amplified with a universal primer pair 338F (ACTCCTACGGGAGGCAGCAG) and 806R (GGACTACHVGGGTWTCTAAT). The PCR-amplified production was purified and quantified to construct a sequencing library that was sequenced on the Illumina MiSeq platform (PE300, Illumina Inc., San Diego, CA, USA) at Shanghai Majorbio Technology Co., Ltd. The data were analyzed on the Majorbio Cloud Platform.

The raw paired-end reads of 16S rRNA gene sequencing were processed and optimized using the Quantitative Insights into Microbial Ecology 2 (QIIME 2, version 2024.5). The representative sequence and abundance information of amplicon sequence variants (ASVs) was obtained using the DADA2 package for filtering, dereplication, identification of chimera, and merging paired-end reads^[12]^. The taxonomy of each ASV representative sequence was annotated using the Silva database (version 138). The alpha diversity indices were calculated by using Mothur (version 1.30.2) based on the ASVs information. The principal coordinate analysis (PCoA) of ASVs based on the weighted UniFrac distance was plotted and analyzed with the vegan package (version 2.4.3) in R (version 3.3.1). The composition of microbial community at the phylum and genus levels was visualized with bar charts by the ggplot2 package (version 2.2.1). The heatmap and cluster tree were analyzed and graphed by pheatmap package (version 1.0.8). Spearman’s correlation analysis was performed on the genera associated with clinical parameters or SCFAs. The Linear discriminant analysis effect size (LEfSe) was used to identify taxa that have significantly differential effects on the sample grouping, which were ranked by linear discriminant analysis (LDA) scores. A random forest model was implemented using the RandomForest package in R to identify microbial signatures associated with inulin intervention in patients with PCOS. Model performance was optimized through receiver operating characteristic (ROC) analysis with optimal classification thresholds.

**Metagenomic sequencing and data analysis**

Microbial genome of fecal samples from mice in Trial-3 were further identified through metagenomic sequencing. In fecal transplantation experiments, the gut microbiome of donors and recipients were defined using shallow metagenomic sequencing , which provided taxonomic and functional annotation at species-level^[13]^. Total genomic DNA from fecal samples was extracted according to E.Z.N.A.® Soil DNA Kit (Omega Bio-tek, Norcross, GA, U.S.) instruction. After assessing of the purity and quality of the extracted DNA, it was fragmented to an average size of approximately 300 bp by Covaris M220 (Gene Company Limited, China) for the sequencing library construction, using NEXTflex® Rapid DNA-Seq Kit (Bioo Scientific, Austin, TX, USA) based on the manufacturer's instruction. Metagenomic sequencing was performed on the Illumina NovaSeq (PE150, Illumina Inc.) at Shanghai Honsunbio Technology Co., Ltd.

The raw sequencing data were were cleaned by removing adapters and low-quality reads, followed by filtering of the remaining read pairs against the human genome alignment to eliminate host DNA. The decontaminated reads were analyzed by Kraken2 and Bracken^[14]^ for taxonomic annotation and abundance against the NCBI blast nonredundant protein database^[15]^. The carbohydrate-active enzymes (CAZy) annotation was performed using a HMMscan pipeline against the CAZy database^[16]^. The antibiotic resistance genes (ARGs) annotation was conducted using resistance gene identifier (RGI) software against the comprehensive antibiotic resistance database (CARD)^[17]^. The virulence factor genes (VFGs) was annotated against the virulence factor database (VFDB)^[18]^ using Diamond (version 2.1.8). The protein sequences for formate-tetrahydrofolate ligase, propionyl-CoA:succinate-CoA transferase, and propionate CoA-transferase were retrieved through a text search in the NCBI database^[19]^. Additionally, the sequences for 4Hbt, AtoA, AtoD, Buk, and But were obtained from the butyrate-producing pathways database as described previously^[20]^. Functional profiling was conducted using HUMAnN (the HMP Unified Metabolic Analysis Network, version 3.0) to obtain KEGG pathway information based on the conversion of the Uniref 90 (90% UniProt Reference Clusters)^[15, 21]^. The principal coordinate analysis (PCoA) of species or genes based on the Bray-Curtis distance was conducted and visualized with the vegan package (version 2.4.3) in R (version 3.3.1). The LEfSe was applied to identify discrepant KEGG pathways between different groups with LDA scores of > 2.0.

**Microbial** **Co-abundant group (CAG) network analysis**

To identify the key responsive community to inulin intervention, we performed a CAG network analysis. Species with total abundance of more than 50 and co-occurrence rate of at least 80% among samples were included, and pairwise correlations were calculated using the SparCC algorithm^[22]^. The resulting SparCC correlation matrix was converted into a distance matrix, then Ward’s hierarchical clustering method and PERMANOVA (999 permutations) were used to cluster species into 42 CAGs. A cluster tree was generated, and non-significant clusters (p > 0.001) were iteratively merged into a single CAG by PERMANOVA from the root to terminal nodes. The final CAG network was visualized using Gephi software^[23]^.

**Estrous cycle**

Vaginal smears were performed every morning for ten days. The smears were stained with crystal violet, followed by microscopic evaluation of the predominant cell type to determine the stage of the estrous cycle. The proestrus (P) is dominated by irregular nucleated epithelial cells. The estrus (E) is dominated by lamellar anucleate cornified epithelial cells. The metestrus (M) stage is dominated by cornified epithelial cells and leukocytes. The diestrus (D) is dominated by a mass of leukocytes.

**Glucose- and insulin-tolerance tests**

Mice were fasted for 12 h or 4 h before the intraperitoneal glucose tolerance test (GTT) or intraperitoneal insulin tolerance test (ITT), respectively. Fasting blood glucose levels were first measured, then the mice were injected intraperitoneally with glucose (2.0 g/kg body weight) for the GTT or insulin (1.0 IU/kg body weight) for the ITT, followed by measuring blood glucose levels at 15, 30, 60, 90, and 120 min, using a Accu-Check Performa blood glucose meter (Roche, USA) with blood collected from tail vein.

**Biochemical analysis**

Fresh blood samples of mice were centrifuged at 3000 rpm for 20 min at 4 ℃ and the serum was collected and stored at -80 ℃ till assay. Serum insulin (90080, Crystal Chem, USA), testosterone (RK00724, ABclonal, China), FSH (CSB-E06871m, CUSABIO, China), LH (RK15292, ABclonal), AMH (RK09261, ABclonal), LBP (RK09173, ABclonal), IL-1β (RK 04878, ABclonal), and IL-18 (RK00104, ABclonal) concentrations were measured using different mouse ELISA kits according to each manufacturer’s instructions.

**Short-chain fatty acids (SCFAs) profiling**

SCFAs in the cecum of mice were detected by gas chromatography-mass spectrometry (GC-MS). Twenty mg cecum content was homogenized with 800 μL phosphoric acid (0.5% v/v) solution and ultrasonic for 10 min. Supernatants (200 μL) were transferred into a new tube, following by adding 200 μL of n-butanol solution for SCFAs extraction with vortex for 10 s and ultrasound for 10 min under low temperature. After centrifugation at 13000 r/min for 5 min at 4 °C, the supernatants were collected for subsequent SCFAs measurement using an Agilent 8890B-7000D (Agilent Technologies Inc. CA, UAS). The Masshunter software (version 10.0.707.0) was used to identify SCFAs with the default parameters accompanied by manual inspection, and the concentration of each sample was calculated based on a standard curve. The sum of all types of SCFAs was defined as the total SCFAs^[9]^.

**Histology and immunohistochemistry**

Ex-vivo ovary, peri-ovarian adipose and colon tissues were collected from the mice and fixed in 4% paraformaldehyde solution for 48 h, followed by individually embedded in paraffin. The paraffin blocks of ovary tissues were serially sectioned and peri-ovarian adipose tissues were sectioned in the largest cross-section at a thickness of 5 μm for hematoxylin-eosin staining. The number of cystic follicles and corpora lutea in the ovary were counted under histomorphologically microscopic and confirmed by a pathologist. Adipocyte sizes were assessed using Image J software (version 1.53).

The paraffin blocks of colon tissues were sectioned transversely at 5 mm for immunohistochemistry. Anti-Zo1 pAb (1:200, A0659, ABclonal), anti-Claudin1 mAb (1:200, A11530, ABclonal), anti-Occludin mAb (1:200, A24601, ABclonal), and HRP-conjugated goat anti-rabbit IgG (WH1083, WeiaoBio, China) were applied in the sections separately for immunohistochemistry staining. The staining was revealed by adding 3,3'-diaminobenzidine solution and the reaction was stopped at the same time for all the samples. The sections were then stained by hematoxylin, dehydrated with alcohol and xylene, and sealed. The staining area of each section was quantified using Image J software (version 1.53).

**Real-time quantitative PCR assays**

Total RNA from colon and brown adipose tissues in mice was isolated using the Trizol reagent (RK30129, ABclonal). The reverse transcription of RNA to cDNA was conducted using the ABScript III RT Master Mix (RK20428, ABclonal). The real-time qPCR was performed using SYBR Green Fast mix (RK21206, ABclonal) in QuantStudio 5 PCR system (ABI, USA). The PCR conditions were: 95 °C for 3 min, followed by 45 cycles of 95 °C for 5 s and 60 °C for 34 s. Relative mRNA expression of each gene was analyzed using the 2^−ΔΔCT^ method with *β-actin* as the housekeeping gene.

The primer sequences are as follows:

| Genes | Species | Direction | Sequences (5’→3’) |  |
| --- | --- | --- | --- | --- |
| *β-actin* | Mus musculus | Forward | GGCTGTATTCCCCTCCATCG |  |
|  |  | Reverse | CCAGTTGGTAACAATGCCATGT |  |
| *Cldn1* | Mus musculus | Forward | GGGGACAACATCGTGACCG |  |
|  |  | Reverse | AGGAGTCGAAGACTTTGCACT |  |
| *Ocln* | Mus musculus | Forward | TTGAAAGTCCACCTCCTTACAGA |  |
|  |  | Reverse | CCGGATAAAAAGAGTACGCTGG |  |
| *Zo1* | Mus musculus | Forward | GCCGCTAAGAGCACAGCAA |  |
|  |  | Reverse | TCCCCACTCTGAAAATGAGGA |  |
| *Ucp1* | Mus musculus | Forward | AGGCTTCCAGTACCATTAGGT |  |
|  |  | Reverse | CTGAGTGAGGCAAAGCTGATTT |  |
| *Pgc1a* | Mus musculus | Forward | TATGGAGTGACATAGAGTGTGCT |  |
|  |  | Reverse | CCACTTCAATCCACCCAGAAAG |  |
| *Cited1* | Mus musculus | Forward | AACCTTGGAGTGAAGGATCGC |  |
|  |  | Reverse | GTAGGAGAGCCTATTGGAGATGT |  |
| *Ppara* | Mus musculus | Forward | AGAGCCCCATCTGTCCTCTC |  |
|  |  | Reverse | ACTGGTAGTCTGCAAAACCAAA |  |
| *Dio2* | Mus musculus | Forward | AATTATGCCTCGGAGAAGACCG |  |
|  |  | Reverse | GGCAGTTGCCTAGTGAAAGGT |  |
| *Ffar2* | Mus musculus | Forward | CTTGATCCTCACGGCCTACAT |  |
|  |  | Reverse | CCAGGGTCAGATTAAGCAGGAG |  |
| *Ffar3* | Mus musculus | Forward | TTCTGAGCGTGGCCTATCCA |  |
|  |  | Reverse | AGACTACACTGACCAGACCAG |  |
| *Il18* | Mus musculus | Forward | GACTCTTGCGTCAACTTCAAGG | |
|  |  | Reverse | CAGGCTGTCTTTTGTCAACGA | |
| *Il1b* | Mus musculus | Forward | GCAACTGTTCCTGAACTCAACT | |
|  |  | Reverse | ATCTTTTGGGGTCCGTCAACT | |
| *Lbp* | Mus musculus | Forward | GTCGTGGGCAGTACGAGTTTC | |
|  |  | Reverse | CTCGGACACCGATGGAAGA | |
| *Nlrp3* | Mus musculus | Forward | ATTACCCGCCCGAGAAAGG | |
|  |  | Reverse | TCGCAGCAAAGATCCACACAG | |
| *Nlrp1* | Mus musculus | Forward | GGTGGTGTGAAGATGTTGTGT | |
|  |  | Reverse | TCCATGTTCATCGTAGGGACC | |
| *Nlrc4* | Mus musculus | Forward | ATCGTCATCACCGTGTGGAG | |
|  |  | Reverse | GCCAGACTCGCCTTCAATCA | |

**Western blot**

Ovarian tissues of mice were grinded into homogenate in RIPA buffer (PC101, Yamei, China) containing protease and phosphatase inhibitors (P002, NCM Biotech, China). The extracted ovarian proteins were separated through SDS-polyacrylamide gel electrophoresis and transferred onto polyvinylidene difluoride membranes. The membranes were blocked with 5% milk and then incubated with specific antibodies overnight at 4 °C. The antibodies were anti β-actin (AC038, ABclonal), TLR4 (14358, CST, USA), MyD88 (A21905, ABclonal), NF-kB p65/ ser536 (A22331, ABclonal), Phospho-NF-κB p65/ser536 (3033, CST), NLRP3 (A24294, ABclonal), ASC (67824, CST), Cleaved Caspase-1 (89332, CST), and Cleaved Gasdermin D (10137, CST). The following day, the membranes were incubated with HRP-conjugated goat anti-rabbit IgG (A0208, Beyotime, China) for one hour at room temperature. The bands of proteins were visualized with a highly sensitive chemiluminescence test kit (SQ201, Yamei) in imaging system (Tanon, China). Band intensities were analyzed semi-quantitatively using Image J software (version 1.53) to quantify gray values and normalized by β-actin intensity levels.

**Statistical analysis**

In animal studies, data are shown as the mean ± SEM and statistical analyses were performed using GraphPad Prism software (version 9.0) with P values of <0.05 considered significant. Differences between three groups were analyzed using one-way ANOVA with Tukey’s multiple comparisons test. Student’s t test was applied to compare the difference between two groups. In human studies, SPSS (version 22.0) was used for variables with normal distribution, data are shown as the mean ± SD and analyzed by paired Student’s t test; for variables without normal distribution, data are shown as the median with IQR and analyzed by Wilcoxon signed-rank test.

**Resource availability**

The 16S rRNA gene sequencing data has been submitted to the NCBI Sequence Read Archive (SRA) database with the accession number PRJNA1130141 and PRJNA1130144. The metagenomic sequencing data are available in the SRA database at NCBI with the accession number PRJNA1221223 and PRJNA1131085. Any additional data supporting the findings of this study are available from the lead contact (chenmiaoxin@51mch.com) upon request.

**References**

[1] *Human reproduction* **2004**, *19* (1), 41, <https://doi.org/10.1093/humrep/deh098>.

[2] R. Ziaei, Z. Shahshahan, H. Ghasemi‐Tehrani, Z. Heidari, M. S. Nehls, R. Ghiasvand, *Food Science & Nutrition* **2023**, *12* (3), 2016, https://doi.org/10.1002/fsn3.3899.

[3] R. Ziaei, Z. Shahshahan, H. Ghasemi-Tehrani, Z. Heidari, R. Ghiasvand, *Clinical endocrinology* **2022**, *97* (3), 319, https://doi.org/10.1111/cen.14712.

[4] S. G. Shamasbi, S. Ghanbari-Homayi, M. Mirghafourvand, *European journal of nutrition* **2020**, *59* (2), 433, https://doi.org/10.1007/s00394-019-02033-1.

[5] X. Qi, C. Yun, L. Sun, J. Xia, Q. Wu, Y. Wang, L. Wang, Y. Zhang, X. Liang, L. Wang, F. J. Gonzalez, A. D. Patterson, H. Liu, L. Mu, Z. Zhou, Y. Zhao, R. Li, P. Liu, C. Zhong, Y. Pang, C. Jiang, J. Qiao, *Nature medicine* **2019**, *25* (8), 1225, <https://doi.org/10.1038/s41591-019-0509-0>.

[6] H. Lai, X. Jia, Q. Yu, C. Zhang, J. Qiao, Y. Guan, J. Kang, *Biology of reproduction* **2014**, *91* (5), <https://doi.org/10.1095/biolreprod.114.120063>.

[7] J. Rodriguez, S. Hiel, A. M. Neyrinck, T. Le Roy, S. A. Potgens, Q. Leyrolle, B. D. Pachikian, M. A. Gianfrancesco, P. D. Cani, N. Paquot, M. Cnop, N. Lanthier, J. P. Thissen, L. B. Bindels, N. M. Delzenne, *Gut* **2020**, *69* (11), 1975, https://doi.org/10.1136/gutjnl-2019-319726.

[8] S. Hiel, A. M. Neyrinck, J. Rodriguez, B. D. Pachikian, C. Bouzin, J.-P. Thissen, P. D. Cani, L. B. Bindels, N. M. Delzenne, *Nutrients* **2018**, *10* (5), https://doi.org/10.3390/nu10050532.

[9] X. Yuan, R. Wang, B. Han, C. Sun, R. Chen, H. Wei, L. Chen, H. Du, G. Li, Y. Yang, X. Chen, L. Cui, Z. Xu, J. Fu, J. Wu, W. Gu, Z. Chen, X. Fang, H. Yang, Z. Su, J. Wu, Q. Li, M. Zhang, Y. Zhou, L. Zhang, G. Ji, F. Luo, *Nat Commun* **2022**, *13* (1), 6356, https://doi.org/10.1038/s41467-022-33656-4.

[10] C. Zhang, A. Yin, H. Li, R. Wang, G. Wu, J. Shen, M. Zhang, L. Wang, Y. Hou, H. Ouyang, Y. Zhang, Y. Zheng, J. Wang, X. Lv, Y. Wang, F. Zhang, B. Zeng, W. Li, F. Yan, Y. Zhao, X. Pang, X. Zhang, H. Fu, F. Chen, N. Zhao, B. R. Hamaker, L. C. Bridgewater, D. Weinkove, K. Clement, J. Dore, E. Holmes, H. Xiao, G. Zhao, S. Yang, P. Bork, J. K. Nicholson, H. Wei, H. Tang, X. Zhang, L. Zhao, *EBioMedicine* **2015**, *2* (8), 968, https://doi.org/10.1016/j.ebiom.2015.07.007.

[11] S. C. Bokoliya, Y. Dorsett, H. Panier, Y. Zhou, *Frontiers in cellular and infection microbiology* **2021**, *11*, https://doi.org/10.3389/fcimb.2021.711055.

[12] B. J. Callahan, P. J. McMurdie, M. J. Rosen, A. W. Han, A. J. A. Johnson, S. P. Holmes, *Nature Methods* **2016**, *13* (7), 581, https://doi.org/10.1038/nmeth.3869.

[13] B. Hillmann, G. A. Al-Ghalith, R. R. Shields-Cutler, Q. Zhu, D. M. Gohl, K. B. Beckman, R. Knight, D. Knights, J. F. Rawls, *mSystems* **2018**, *3* (6), e00069, https://doi.org/10.1128/mSystems.00069-18.

[14] A. Mannion, A. Sheh, Z. Shen, J. Dzink-Fox, M. B. Piazuelo, K. T. Wilson, R. Peek, J. G. Fox, *Gut Microbes* **2023**, *15* (1), 2186677, https://doi.org/10.1080/19490976.2023.2186677.

[15] M. R. Stothart, P. D. McLoughlin, J. Poissant, *Molecular Ecology Resources* **2022**, *23* (3), 549, https://doi.org/10.1111/1755-0998.13713.

[16] E. Drula, M.-L. Garron, S. Dogan, V. Lombard, B. Henrissat, N. Terrapon, *Nucleic Acids Research* **2022**, *50* (D1), D571, <https://doi.org/10.1093/nar/gkab1045>.

[17] B. P. Alcock, W. Huynh, R. Chalil, K. W. Smith, Amogelang R. Raphenya, M. A. Wlodarski, A. Edalatmand, A. Petkau, S. A. Syed, K. K. Tsang, S. J. C. Baker, M. Dave, Madeline C. McCarthy, K. M. Mukiri, J. A. Nasir, B. Golbon, H. Imtiaz, X. Jiang, K. Kaur, M. Kwong, Z. C. Liang, K. C. Niu, P. Shan, J. Y. J. Yang, Kristen L. Gray, G. R. Hoad, B. Jia, T. Bhando, Lindsey A. Carfrae, Maya A. Farha, S. French, R. Gordzevich, K. Rachwalski, Megan M. Tu, E. Bordeleau, D. Dooley, E. Griffiths, H. L. Zubyk, E. D. Brown, F. Maguire, Robert G. Beiko, W. W. L. Hsiao, F. S. L. Brinkman, G. Van Domselaar, A. G. McArthur, *Nucleic Acids Research* **2023**, *51* (D1), D690, https://doi.org/10.1093/nar/gkac920.

[18] B. Liu, D. Zheng, S. Zhou, L. Chen, J. Yang, *Nucleic Acids Research* **2022**, *50* (D1), D912, https://doi.org/10.1093/nar/gkab1107.

[19] L. Zhao, F. Zhang, X. Ding, G. Wu, Y. Y. Lam, X. Wang, H. Fu, X. Xue, C. Lu, J. Ma, L. Yu, C. Xu, Z. Ren, Y. Xu, S. Xu, H. Shen, X. Zhu, Y. Shi, Q. Shen, W. Dong, R. Liu, Y. Ling, Y. Zeng, X. Wang, Q. Zhang, J. Wang, L. Wang, Y. Wu, B. Zeng, H. Wei, M. Zhang, Y. Peng, C. Zhang, *Science* **2018**, *359* (6380), 1151, https://doi.org/10.1126/science.aao5774.

[20] M. Vital, A. C. Howe, J. M. Tiedje, M. A. Moran, *mBio* **2014**, *5* (2), https://doi.org/10.1128/mBio.00889-14.

[21] C. H. Wu, *Nucleic Acids Research* **2006**, *34* (90001), D187, https://doi.org/10.1093/nar/gkj161.

[22] C. von Mering, J. Friedman, E. J. Alm, *PLoS Computational Biology* **2012**, *8* (9), https://doi.org/10.1371/journal.pcbi.1002687.

[23] P. Wang, X. Yang, L. Zhang, S. Sha, J. Huang, J. Peng, J. Gu, J. A. Pearson, Y. Hu, H. Zhao, F. S. Wong, Q. Wang, L. Wen, *Nature Communications* **2024**, *15* (1), https://doi.org/10.1038/s41467-024-48611-8.
